# THSD7B promotes tumor progression and is associated with prognosis in gastric adenocarcinoma

**DOI:** 10.1371/journal.pone.0351545

**Published:** 2026-06-12

**Authors:** Xinying Quan, Wei Cheng, Yao Pu, Hong Deng

**Affiliations:** 1 Department of Blood Transfusion, Guizhou Branch of Beijing Jishuitan Hospital, Guiyang, Guizhou, P.R. China; 2 Hematology Department, Affiliated Hospital of Guizhou Medical University, Guiyang, ‌‌Guizhou, P.R. China; 3 Department of Pharmacy, Guiyang Second People’s Hospital, Guiyang, Guizhou, P.R. China; 4 Laboratory Department, People’s Hospital of Baiyun District, Baiyun, ‌‌P.R. China; Longgang Otorhinolaryngology Hospital & Shenzhen Key Laboratory of Otorhinolaryngology, Shenzhen Institute of Otorhinolaryngology, CHINA

## Abstract

THSD7B (thrombospondin type-1 domain-containing 7B) has been implicated in several malignancies; however, its role in gastric adenocarcinoma remains unclear. This study aimed to investigate the expression pattern, clinical significance, and biological function of THSD7B in gastric adenocarcinoma. Public datasets from The Cancer Genome Atlas (TCGA) and Gene Expression Omnibus (GEO) were analyzed to evaluate THSD7B expression and its association with clinical outcomes. Functional enrichment analysis was performed to explore potential biological processes. In vitro assays, including cell proliferation, colony formation, wound healing, and Transwell invasion, were conducted following THSD7B knockdown or overexpression in gastric cancer cell lines. In addition, a xenograft model was established to assess tumor growth in vivo. THSD7B expression was significantly elevated in gastric adenocarcinoma tissues compared with normal controls and was associated with patient survival. Functional analyses suggested that THSD7B-related genes were mainly enriched in cell adhesion and cytoskeleton-associated processes. In vitro experiments showed that THSD7B knockdown suppressed cell proliferation, migration, and invasion, whereas overexpression produced the opposite effects. Consistent with these findings, THSD7B modulation was accompanied by alterations in adhesion-related signaling molecules and phenotype-associated protein expression. In vivo, THSD7B promoted tumor growth in xenograft models. In conclusion, THSD7B is associated with tumor progression and clinical outcomes in gastric adenocarcinoma and may be involved in the regulation of cell motility-related processes. These findings suggest that THSD7B may serve as a potential biomarker in gastric cancer.

## Introduction

Gastric cancer remains a major cause of cancer-related morbidity and mortality worldwide. Despite improvements in diagnostic strategies and therapeutic approaches, the prognosis of patients with advanced disease remains unsatisfactory [1–3]. Increasing evidence suggests that gastric cancer progression is driven by a combination of tumor-intrinsic alterations and interactions with the surrounding microenvironment, highlighting the need to identify additional molecules associated with these processes [4].

Cell adhesion and cytoskeleton-associated signaling are essential regulators of tumor cell behavior [5,6]. These processes influence cellular proliferation, migration, and invasion, and are frequently altered during tumor progression [7]. Adhesion-related signaling pathways, including those involving focal adhesion kinase (FAK) and Src family kinases, have been reported to participate in the regulation of cell motility and phenotypic plasticity [8–10]. Although these pathways have been studied in multiple tumor types, the upstream factors contributing to their dysregulation in gastric cancer remain incompletely understood.

THSD7B (thrombospondin type-1 domain-containing 7B) encodes a protein containing thrombospondin type-1 repeat domains, which are known to participate in diverse biological processes, including cell motility, extracellular interactions, and angiogenesis. Consistent with this, THSD7B has been predicted to be involved in cytoskeleton organization and cell adhesion-related processes [11,12]. Previous studies have suggested that proteins in this family may participate in tumor-associated biological behaviors; however, the role of THSD7B in gastric cancer has not been well characterized [13,14]. Current evidence regarding THSD7B is limited, and its potential involvement in tumor progression and related signaling remains to be clarified.

In the present study, we aimed to investigate the expression pattern and clinical relevance of THSD7B in gastric cancer using publicly available datasets, followed by experimental validation in vitro and in vivo. Functional enrichment analysis was used to provide an overview of biological processes associated with THSD7B-related genes. Based on these observations, we further evaluated whether THSD7B is associated with malignant cellular behaviors and selected signaling pathways. This study was designed to provide an integrated assessment of THSD7B in gastric cancer while maintaining a cautious interpretation of its potential biological roles.

## Materials and methods

### Data acquisition and preprocessing

Gene expression profiles and corresponding clinical information were obtained from The Cancer Genome Atlas (TCGA) database. Pan-cancer analyses were performed using TCGA-PANCANCER datasets, while gastric adenocarcinoma-specific analyses were conducted using the TCGA stomach adenocarcinoma cohort (TCGA-STAD). TPM-format RNA-sequencing data processed using the STAR pipeline were used for downstream analyses. For external validation, the microarray dataset GSE13861 was downloaded from the Gene Expression Omnibus (GEO) database. Expression values were transformed using log2(value + 1) where appropriate. Samples lacking complete clinical information were excluded from survival-related analyses.

### Differential expression and survival analysis

The expression levels of THSD7B between tumor and normal tissues were compared using the Wilcoxon rank-sum test. Associations between THSD7B expression and clinicopathological variables were evaluated using non-parametric tests. Kaplan–Meier survival analysis was performed to compare overall survival and disease free survical differences between groups using the log-rank test. Time-dependent receiver operating characteristic (ROC) curves were generated to evaluate the predictive performance of THSD7B. Pan-cancer exploratory survival analyses were performed using TCGA-PANCANCER datasets.

### Cox regression analysis

To further evaluate the prognostic relevance of THSD7B in gastric adenocarcinoma, univariate and multivariate Cox proportional hazards regression analyses were performed using TCGA-STAD clinical data. Overall survival (OS) was used as the prognostic endpoint. Patients were divided into high- and low-expression groups according to the median THSD7B expression level. Variables meeting the predefined significance threshold in univariate analysis were subsequently included in the multivariate Cox regression model. Hazard ratios (HRs) and 95% confidence intervals (CIs) were calculated using the R packages “survival” (version 3.3−1) and “rms” (version 6.3−0) in R software (version 4.2.1). Proportional hazards assumptions were assessed using the survival package. A two-sided P value < 0.05 was considered statistically significant.

### Functional enrichment analysis

Genes correlated with THSD7B expression were identified based on correlation analysis. These genes were subsequently subjected to Gene Ontology (GO) and Kyoto Encyclopedia of Genes and Genomes (KEGG) enrichment analyses using the R environment. Gene set enrichment analysis (GSEA) was performed to explore potential biological processes associated with THSD7B expression. Enrichment results were visualized using standard R packages.

### Cell culture and transfection

AGS, SNU-1, and MKN-45 cells were purchased from Procell Life Science & Technology Co., Ltd. (Wuhan, China). Cells were maintained in RPMI-1640 medium supplemented with 10% fetal bovine serum at 37°C in a humidified incubator with 5% CO₂. For knockdown experiments, cells were transfected with THSD7B siRNA (Santa Cruz Biotechnology, sc-94278) or corresponding negative control siRNA. According to the manufacturer, THSD7B siRNA (h) consists of a pool of 3–5 target-specific 19–25 nt siRNA duplexes against human THSD7B. For overexpression experiments, cells were transfected with plasmids encoding THSD7B or empty vector controls. Transfections were performed using Lipofectamine 3000 (Invitrogen) according to the manufacturer’s instructions.

### FAK activation assay

For pharmacological rescue experiments, THSD7B-silenced gastric cancer cells were treated with the FAK activator M64HCI. Briefly, cells were transfected with si-THSD7B or negative control siRNA as described above. After transfection, cells were incubated with M64HCI (20μM) for 24 h before protein extraction or functional assays. Cells treated with vehicle under the same conditions were used as controls. Western blotting was performed to evaluate the levels of p-FAK, FAK, p-SRC, SRC, and phenotype-associated proteins.

### Quantitative real-time PCR (qPCR)

Total RNA was extracted using TRIzol reagent (Invitrogen) and reverse-transcribed into cDNA using a commercial reverse transcription kit. Quantitative real-time PCR was conducted using SYBR Green Master Mix on a real-time PCR system. Relative expression levels were calculated using the 2^ − ΔΔCt method with GAPDH as the internal reference. The primer sequences used for qPCR were as follows: THSD7B forward, 5′-GCACCTTCACTGCTTGGTCCAA-3′ and reverse, 5′-GGGTAAAGGTCAGAATCCTGGC-3′; β-actin/ACTB forward, 5′-CATGTACGTTGCTATCCAGGC-3′ and reverse, 5′-CTCCTTAATGTCACGCACGAT-3′.

### Cell proliferation assay

Cell proliferation was assessed using the Cell Counting Kit-8 (CCK-8, Dojindo). Transfected cells were seeded into 96-well plates, and absorbance at 450 nm was measured at indicated time points following incubation with CCK-8 reagent.

### Colony formation assay

Cells were plated in 6-well plates at low density and cultured for approximately 10–14 days. Colonies were fixed with paraformaldehyde, stained with crystal violet, and counted under a microscope.

### Wound healing assay

Cells were seeded into 6-well plates and allowed to reach near confluence. A linear scratch was created using a sterile pipette tip, and detached cells were removed by washing with PBS. Images were captured at 0 h and at designated time points to evaluate cell migration.

### Transwell invasion assay

Cell invasion assays were performed using Transwell chambers (Corning) pre-coated with Matrigel. Cells were seeded in serum-free medium in the upper chamber, while medium containing serum was added to the lower chamber. After incubation, cells that invaded to the lower surface were fixed, stained, and counted.

### Western blot analysis

Total protein was extracted using RIPA lysis buffer supplemented with protease and phosphatase inhibitors. Protein concentrations were determined using a BCA assay. Equal amounts of protein were separated by SDS-PAGE and transferred onto PVDF membranes. Membranes were blocked with 5% non-fat milk and incubated with primary antibodies overnight at 4°C. The primary antibodies used were as follows: THSD7B (Santa Cruz Biotechnology, sc-248815, 1:500), phospho-FAK (Proteintech, 83933–1-RR, 1:1000), FAK (Proteintech, 12636–1-AP, 1:1000), phospho-SRC (Proteintech, 80706–2-RR, 1:1000), SRC (Proteintech, 11097–1-AP, 1:1000), E-cadherin (Proteintech, 20874–1-AP, 1:2000), N-cadherin (Proteintech, 87073–2-RR, 1:1000), and Vimentin (Proteintech, 10366–1-AP, 1:2000). After incubation with appropriate secondary antibodies, protein bands were visualized using enhanced chemiluminescence (ECL). Western blot quantification was performed by densitometric analysis. Total protein levels were normalized to β-actin, whereas phosphorylated protein levels were normalized to the corresponding total protein. The NC group was set to 1, and relative expression levels in other groups were presented as fold changes relative to NC.

### Xenograft tumor model

All animal experiments were conducted in accordance with institutional guidelines. BALB/c nude mice (female, 4–6 weeks old) were purchased from Wuhan Shulaibao Biotechnology Co., Ltd. (Wuhan, China). Mice were randomly assigned to experimental groups, with five mice in each group (n = 5 per group). Gastric cancer cells transfected with control or THSD7B-targeting constructs were subcutaneously injected into the flank of mice to establish xenograft tumors. Tumor growth was monitored at regular intervals using a caliper, and tumor volume was calculated as (length × width²) / 2. Tumor measurements were performed by investigators blinded to group allocation where possible. No animals were excluded from the final analysis unless they met predefined humane endpoint criteria. No unexpected animal deaths occurred during the experiment. Endpoint tumor weights were compared using Student’s t-test, and tumor growth curves were analyzed using two-way ANOVA. For anesthesia, mice were anesthetized with isoflurane inhalation (2–3% for induction and 1–2% for maintenance) when necessary. At the end of the experiment, mice were euthanized by CO₂ inhalation followed by cervical dislocation to ensure death. Tumors were then excised and weighed.

### Statistical analysis

All statistical analyses were performed using R software and GraphPad Prism. Data are presented as mean ± standard deviation (SD). Data distribution was assessed using the Shapiro–Wilk test where appropriate. For comparisons between two groups, Student’s t-test was used for normally distributed data, while the Mann–Whitney U test was used for non-normally distributed data. Comparisons among multiple groups were performed using one-way ANOVA followed by Tukey’s post hoc test. Tumor growth curves were analyzed using two-way ANOVA. Survival differences were assessed using the log-rank test, and Cox regression analysis was used to evaluate prognostic factors. A two-sided P value < 0.05 was considered statistically significant.

### Ethics statement

All animal experiments were reviewed and approved by the Animal Ethics Committee of Guizhou Branch of Beijing Jishuitan Hospital (Approval No. KT20260330−01). All procedures were conducted in accordance with institutional guidelines for the care and use of laboratory animals. Animals used in this study were obtained from a licensed laboratory animal supplier (Wuhan Shulaibao Biotechnology Co., Ltd., Wuhan, China), and no privately owned animals were involved.

## Results

### Pan-cancer expression profile and prognostic relevance of THSD7B

**Fig 1:**
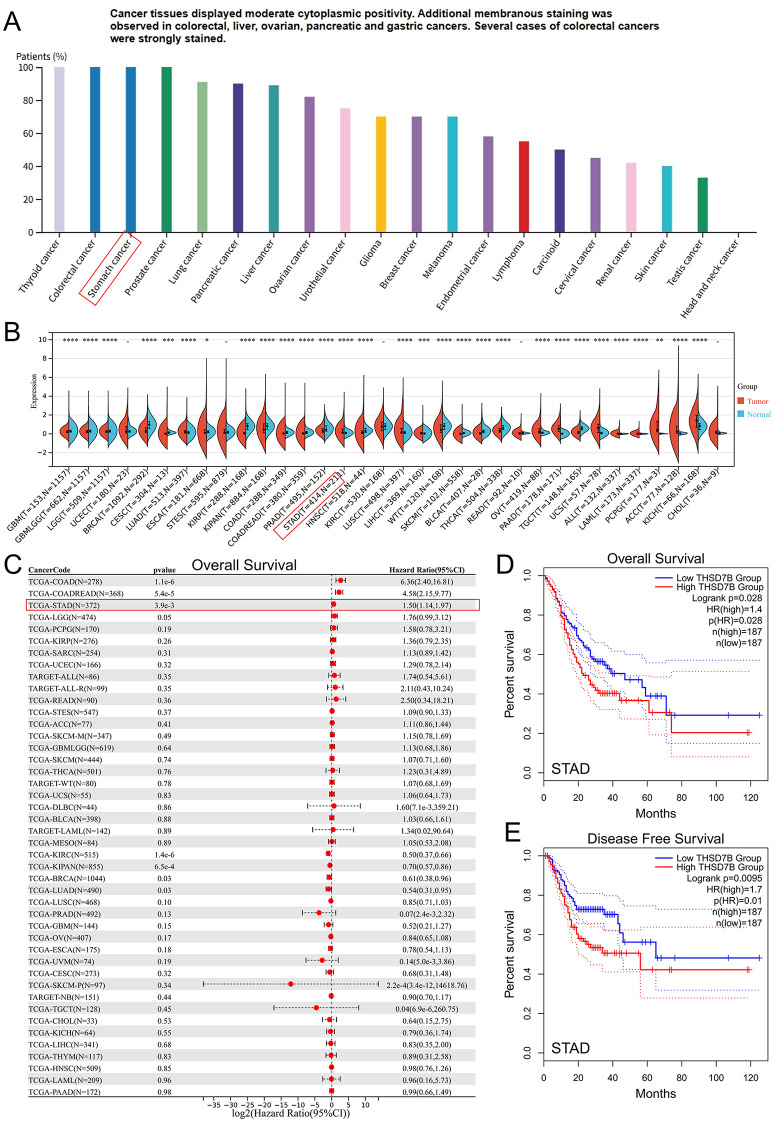
Expression pattern and prognostic significance of THSD7B across cancers. **(A)** Immunohistochemical staining of THSD7B protein across multiple human tumor types obtained from publicly available datasets. Representative staining patterns in different cancers are shown. **(B)** Comparison of THSD7B mRNA expression between tumor and normal tissues across TCGA cancer types. Each dot represents an individual sample. (C) exploratory pan-cancer overview: forest plot showing the association between THSD7B expression and overall survival in different cancers based on TCGA- PANCANCER cohorts. Hazard ratios (HR) and 95% confidence intervals (CI) are indicated. **(D)** Kaplan–Meier analysis of overall survival in patients with stomach adenocarcinoma (STAD) stratified by THSD7B expression levels. **(E)** Kaplan–Meier analysis of disease-free survival in STAD patients according to THSD7B expression. *P < 0.05, **P < 0.01, ***P < 0.005, ****P < 0.001.

THSD7B protein expression was assessed across multiple tumor types using immunohistochemistry data (Fig 1A). Detectable cytoplasmic staining was observed in a broad range of cancers, including gastric cancer, indicating that THSD7B is expressed in diverse tumor tissues. We next evaluated THSD7B mRNA expression across The Cancer Genome Atlas (TCGA) cohorts (Fig 1B). Pan-cancer exploratory survival analyses were also performed using TCGA-PANCANCER datasets. Compared with corresponding normal tissues, THSD7B expression exhibited variable patterns among different tumor types. In stomach adenocarcinoma (STAD), THSD7B was modestly but consistently upregulated relative to normal gastric tissues, indicating dysregulation at the transcriptional level. To further assess the clinical significance of THSD7B, we performed pan-cancer survival analysis (Fig 1C). Elevated THSD7B expression was associated with unfavorable overall survival in several cancer types, including STAD, where a statistically significant hazard ratio was observed. This finding suggests that THSD7B may serve as a potential prognostic indicator in gastric cancer. Kaplan–Meier survival analysis was subsequently conducted specifically in the STAD cohort (Fig 1D–E). Patients with higher THSD7B expression exhibited reduced overall survival compared with those in the low-expression group. A similar trend was observed for disease-free survival, although the statistical significance was less pronounced. Collectively, these results indicate that increased THSD7B expression is associated with poorer clinical outcomes in gastric cancer.

### Functional annotation and validation of THSD7B-related signatures in gastric cancer

**Fig 2:**
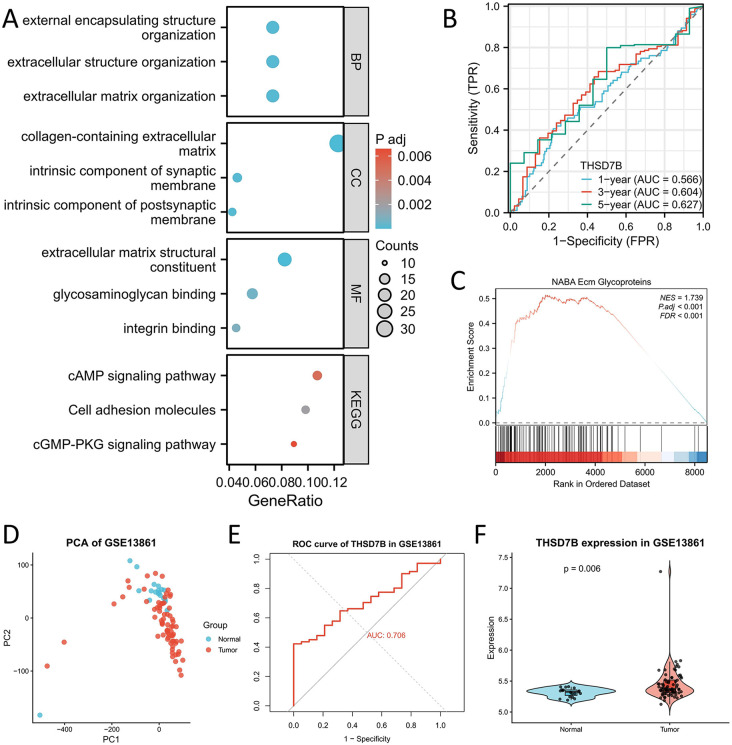
Functional enrichment and external validation of THSD7B in gastric cancer. **(A)** Gene Ontology (GO) and Kyoto Encyclopedia of Genes and Genomes (KEGG) enrichment analysis of THSD7B-related genes. Biological process (BP), cellular component (CC), and molecular function (MF) categories are presented. Bubble size represents gene counts, and color indicates adjusted p values. **(B)** Time-dependent receiver operating characteristic (ROC) curves evaluating the predictive performance of THSD7B expression for 1-, 3-, and 5-year survival. **(C)** Gene set enrichment analysis (GSEA) showing enrichment of the NABA extracellular matrix (ECM) glycoprotein signature in relation to THSD7B expression. **(D)** Principal component analysis (PCA) of samples in GSE13861, colored by tissue type. **(E)** ROC curve assessing the diagnostic performance of THSD7B in GSE13861. **(F)** Comparison of THSD7B expression between tumor and normal tissues in GSE13861.

To further explore the potential biological relevance of THSD7B, functional enrichment analysis was performed based on THSD7B-associated genes. GO analysis indicated that these genes were mainly enriched in processes related to extracellular structure organization, extracellular matrix organization, and external encapsulating structure organization. In the cellular component category, enrichment was observed in collagen-containing extracellular matrix and membrane-related structures, while molecular function terms were mainly associated with extracellular matrix structural constituent, glycosaminoglycan binding, and integrin binding. KEGG pathway analysis further suggested involvement in signaling pathways such as cAMP signaling, cell adhesion molecules, and cGMP–PKG signaling (Fig 2A). To evaluate the potential clinical utility of THSD7B, time-dependent ROC analysis was conducted, showing moderate predictive performance for overall survival at different time points (Fig 2B). In addition, GSEA revealed that the NABA extracellular matrix glycoprotein gene set was significantly enriched in association with THSD7B expression (Fig 2C). External validation was performed using the GSE13861 dataset. PCA demonstrated the distribution pattern of tumor and normal samples based on global gene expression profiles (Fig 2D). ROC analysis indicated that THSD7B exhibited a certain level of discrimination between tumor and normal tissues (Fig 2E). Consistently, expression analysis confirmed differential expression of THSD7B between the two groups (Fig 2F).

### Association of THSD7B with clinicopathological features and prognostic variables in gastric cancer

**Fig 3:**
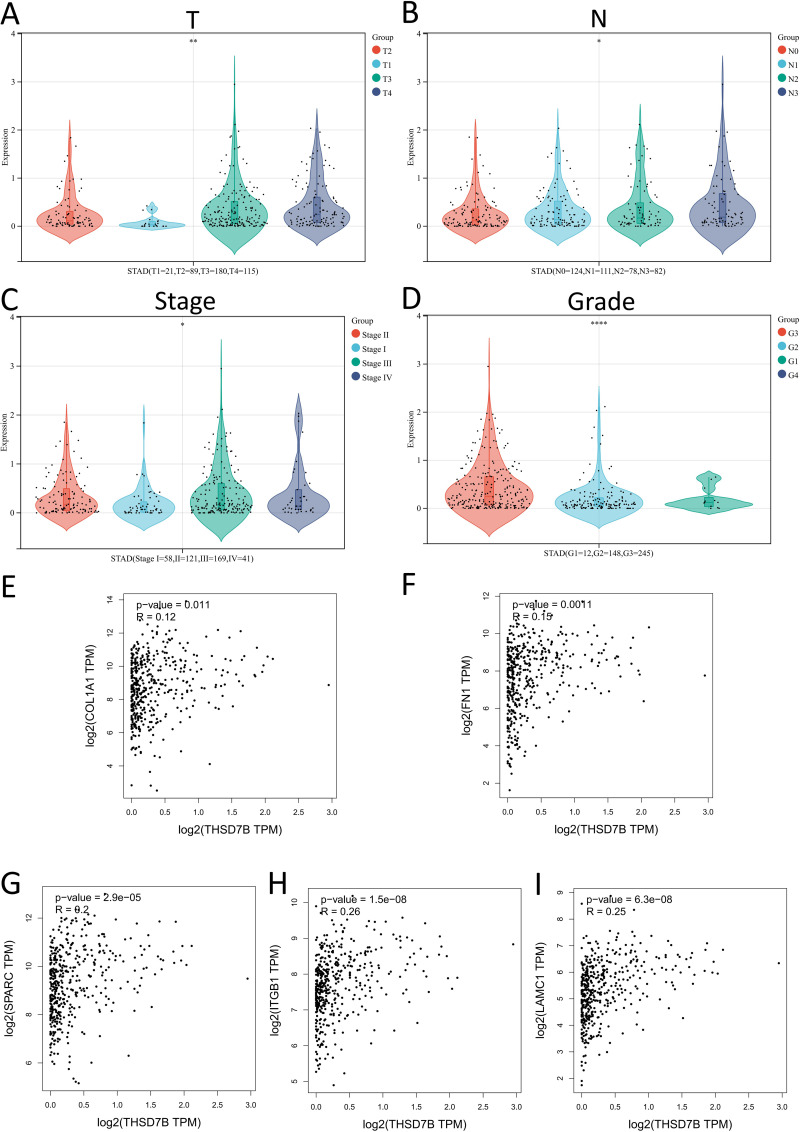
Association of THSD7B with clinicopathological features and related genes in gastric cancer. **(A)** THSD7B expression across different pathological T stages in STAD. **(B)** THSD7B expression across lymph node metastasis (N) stages. **(C)** THSD7B expression across overall pathological stages. **(D)** THSD7B expression across histological grades. (E–I) Correlation analyses between THSD7B expression and representative adhesion- or matrix-related genes, including COL1A1, FN1, SPARC, ITGB1, and LAMC1. Correlation coefficients and P values are indicated‌‌ in each panel. Data are shown as mean ± SD. *P < 0.05, **P < 0.01, ***P < 0.005, ****P < 0.001.

To further investigate the clinical relevance of THSD7B in gastric cancer, its expression was analyzed across different clinicopathological subgroups. THSD7B expression varied among pathological T stages (Fig 3A), lymph node stages (Fig 3B), overall pathological stages (Fig 3C), and histological grades (Fig 3D). These findings suggest that THSD7B expression is related to clinicopathological heterogeneity in STAD. To further evaluate its prognostic relevance, univariate and multivariate Cox regression analyses were performed using TCGA-STAD clinical data. In univariate analysis, high THSD7B expression was associated with poorer survival. After adjustment for clinicopathological variables, high THSD7B expression remained significantly associated with unfavorable outcome, suggesting potential independent prognostic relevance (Table 1). Given the enrichment results, exploratory correlation analyses were performed between THSD7B and representative adhesion- or matrix-related genes. THSD7B expression showed positive correlations with COL1A1, FN1, SPARC, ITGB1, and LAMC1 (Fig 3E–I). However, these correlations were modest and should be interpreted as exploratory associations rather than direct functional evidence.

**Table 1:** Univariate and multivariate Cox regression analyses of clinicopathological variables and THSD7B expression in TCGA-STAD patients.

Characteristics	Total(N)	HR(95% CI) Univariate analysis	P value Univariate analysis	HR(95% CI) Multivariate analysis	P value Multivariate analysis
Age	367				
<= 65	163	Reference		Reference	
> 65	204	1.620 (1.154 - 2.276)	0.005	2.012 (1.387 - 2.919)	< 0.001
Pathologic T stage	362				
T1	18	Reference		Reference	
T2	78	6.725 (0.913 - 49.524)	0.061	4.398 (0.588 - 32.890)	0.149
T3	167	9.548 (1.326 - 68.748)	0.025	5.215 (0.709 - 38.352)	0.105
T4	99	9.634 (1.323 - 70.151)	0.025	4.816 (0.644 - 36.020)	0.126
Pathologic M stage	352				
M0	327	Reference		Reference	
M1	25	2.254 (1.295 - 3.924)	0.004	2.794 (1.518 - 5.141)	< 0.001
Pathologic N stage	352				
N0	107	Reference		Reference	
N1	97	1.629 (1.001 - 2.649)	0.049	1.437 (0.851 - 2.427)	0.175
N2	74	1.655 (0.979 - 2.797)	0.06	1.469 (0.853 - 2.530)	0.165
N3	74	2.709 (1.669 - 4.396)	< 0.001	2.352 (1.393 - 3.973)	0.001
Gender	370				
Female	133	Reference			
Male	237	1.267 (0.891 - 1.804)	0.188		
THSD7B	370				
Low	183	Reference		Reference	
High	187	1.445 (1.039 - 2.009)	0.029	1.697 (1.186 - 2.430)	0.004

HR, hazard ratio; CI, confidence interval; STAD, stomach adenocarcinoma. Overall survival (OS) was used as the prognostic endpoint. Variables meeting the predefined significance threshold in univariate analysis were subsequently included in the multivariate Cox regression model. Hazard ratios (HRs) and corresponding 95% confidence intervals (CIs) were calculated using Cox proportional hazards regression analysis. A two-sided P value < 0.05 was considered statistically significant.

### Effects of THSD7B knockdown on proliferation, migration, and invasion of gastric cancer cells

**Fig 4:**
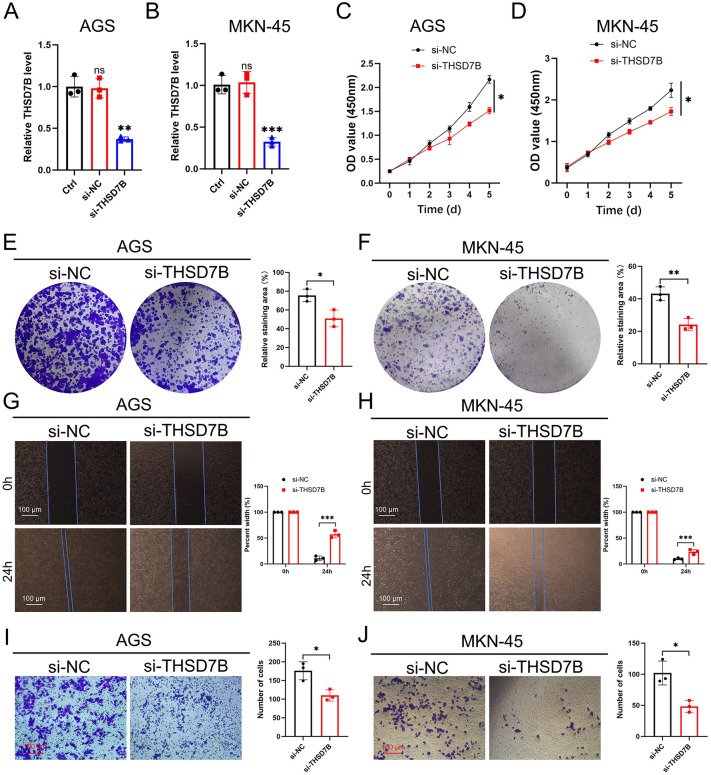
Effects of THSD7B knockdown on proliferation, migration, and invasion of gastric cancer cells. **(A, B)** Relative THSD7B expression levels in AGS and MKN-45 cells following transfection with si-THSD7B, as measured by qPCR. **(C, D)** Cell proliferation curves determined by CCK-8 assay at indicated time points. **(E, F)** Colony formation assays and corresponding quantification in AGS and MKN-45 cells. **(G, H)** Wound healing assays showing cell migration at 0 h and 24 h, with quantification of wound closure. **(I, J)** Transwell invasion assays and quantification of invading cells. Scale: 100μm. Data are shown as mean ± SD. *P < 0.05, **P < 0.01, ***P < 0.001.

To investigate the functional role of THSD7B in gastric cancer, loss-of-function experiments were performed in AGS and MKN-45 cells. As shown in Fig 4A and 4B, transfection with si-THSD7B significantly reduced THSD7B expression compared with control groups, confirming effective knockdown efficiency. Cell proliferation was subsequently assessed using CCK-8 assays. The results showed that THSD7B knockdown led to a reduction in cell growth over time in both AGS and MKN-45 cells (Fig 4C and 4D). Colony formation assays further demonstrated a decrease in clonogenic capacity following THSD7B silencing (Fig 4E and 4F). In addition, wound healing assays indicated a reduction in migratory ability in cells with reduced THSD7B expression (Fig 4G and 4H). Transwell assays were performed to evaluate invasive potential, and fewer invading cells were observed in the si-THSD7B group compared with controls (Fig 4I and 4J).

### Effects of THSD7B overexpression on proliferation, migration, and invasion of gastric cancer cells

**Fig 5:**
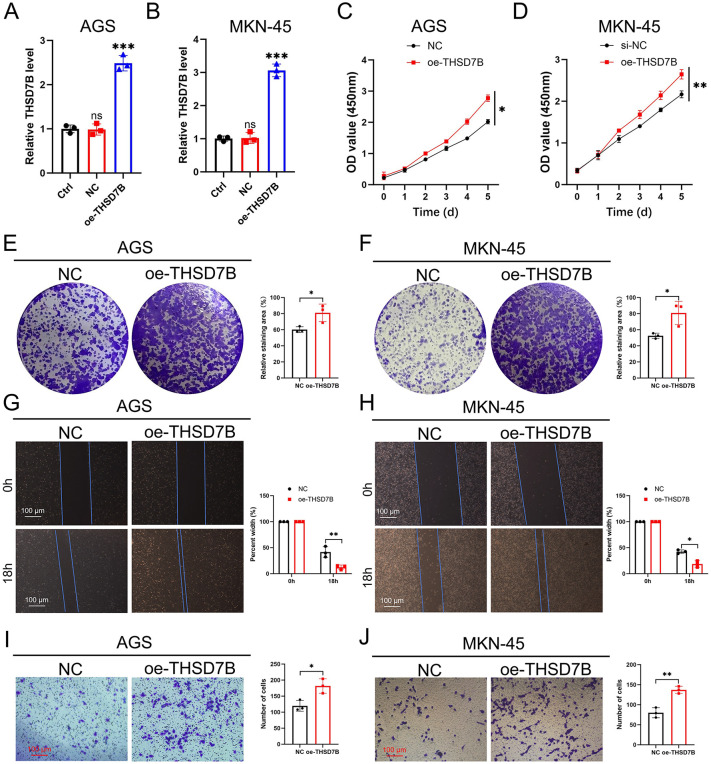
Effects of THSD7B overexpression on proliferation, migration, and invasion of gastric cancer cells. **(A, B)** Relative THSD7B expression levels in AGS and MKN-45 cells after transfection with overexpression plasmid. **(C, D)** Cell proliferation assessed by CCK-8 assay at indicated time points. **(E, F)** Colony formation assays and corresponding quantification. **(G, H)** Wound healing assays at 0 h and 18 h, with quantification of wound closure. **(I, J)** Transwell invasion assays and quantification of invading cells. Scale: 100μm. Data are shown as mean ± SD. *P < 0.05, **P < 0.01, ***P < 0.001.

To further validate the functional role of THSD7B, gain-of-function experiments were performed in AGS and MKN-45 cells. As shown in Fig 5A and 5B, ectopic expression of THSD7B significantly increased its mRNA levels compared with control groups. Cell proliferation assays demonstrated that THSD7B overexpression enhanced cell growth over time in both cell lines (Fig 5C and 5D). Consistently, colony formation assays revealed an increase in clonogenic capacity following THSD7B overexpression (Fig 5E and 5F). Wound healing assays showed that cells overexpressing THSD7B exhibited accelerated closure of the scratch area, indicating enhanced migratory ability (Fig 5G and 5H). In addition, Transwell assays demonstrated that THSD7B overexpression increased the number of invading cells compared with control groups (Fig 5I and 5J).

### THSD7B modulates FAK/SRC-associated signaling and epithelial–mesenchymal phenotype in gastric cancer cells

**Fig 6:**
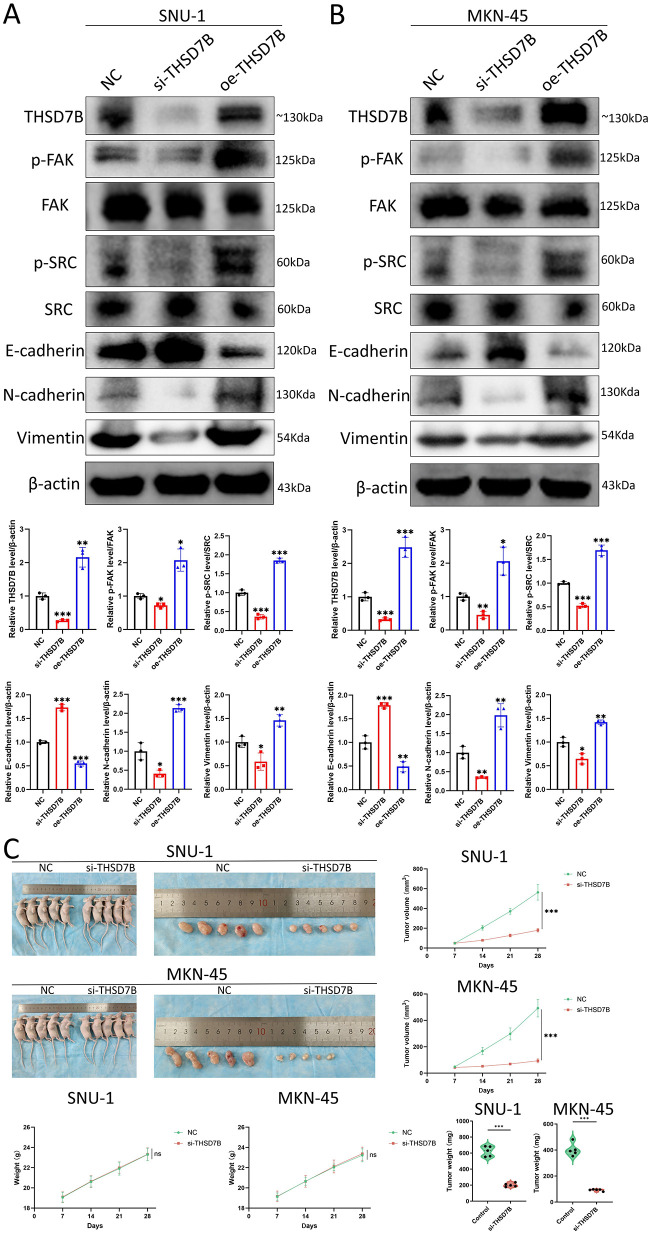
Effects of THSD7B modulation on signaling-related proteins and xenograft tumor growth. **(A–B)** Western blot analysis of THSD7B, p-FAK, FAK, p-SRC, SRC, E-cadherin, N-cadherin, and Vimentin in SNU-1 and MKN-45 cells after THSD7B knockdown or overexpression. β-actin was used as a loading control. Molecular weights are indicated on the right. Relative protein levels were quantified by densitometric analysis. Total proteins were normalized to β-actin, whereas phosphorylated proteins were normalized to the corresponding total protein. The NC group was set to 1. The THSD7B antibody detected a major band at approximately 130 kDa, which was used for quantification because it changed consistently after THSD7B knockdown or overexpression. **(C)** Subcutaneous xenograft models established using SNU-1 and MKN-45 cells with THSD7B knockdown. Representative images of tumor-bearing mice and excised tumors are shown. Tumor growth curves, mouse body weight curves, and endpoint tumor weights are presented. Data are shown as mean ± SD. *P < 0.05, **P < 0.01, ***P < 0.001.

To examine signaling alterations associated with THSD7B expression, western blotting was performed in SNU-1 and MKN-45 cells after THSD7B knockdown or overexpression. The THSD7B antibody detected a major band at approximately 130 kDa, which showed consistent reduction after THSD7B silencing and elevation after THSD7B overexpression. This band was therefore used for quantitative analysis. THSD7B knockdown was accompanied by decreased levels of phosphorylated FAK and phosphorylated SRC, whereas THSD7B overexpression showed the opposite trend. Total FAK and SRC levels remained relatively stable across groups. Changes in phenotype-associated proteins were also observed. THSD7B silencing increased E-cadherin expression and reduced N-cadherin and Vimentin levels, while THSD7B overexpression was associated with decreased E-cadherin and increased N-cadherin and Vimentin. These patterns were observed in both SNU-1 and MKN-45 cells (Fig 6A and B). To further evaluate the in vivo relevance of THSD7B, subcutaneous xenograft models were established using SNU-1 and MKN-45 cells with THSD7B knockdown. Compared with the control group, THSD7B knockdown reduced tumor growth over time and resulted in smaller endpoint tumor weights, whereas mouse body weight showed no significant difference between groups (Fig 6C).

**Fig 7:**
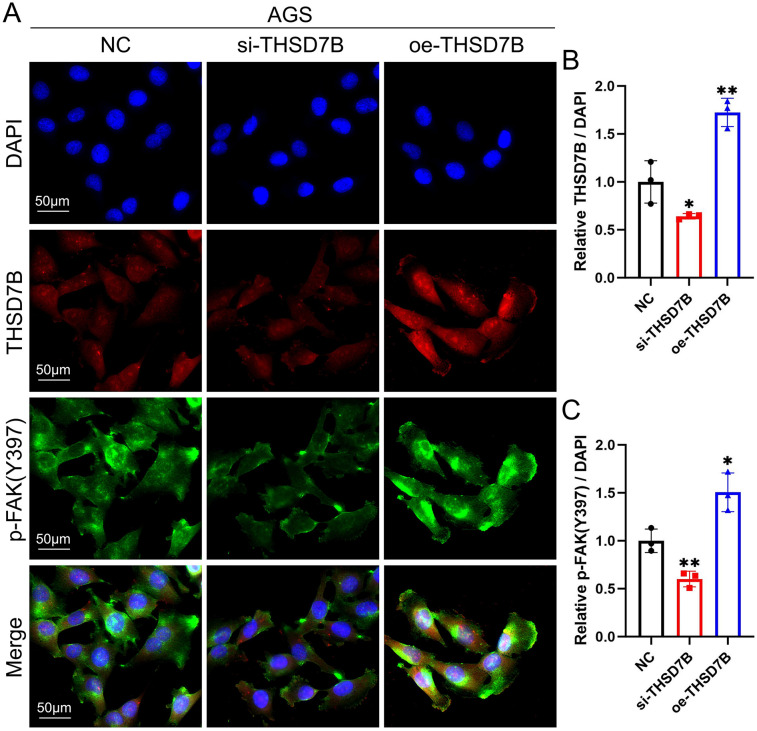
Immunofluorescence analysis of THSD7B and p-FAK(Y397) in gastric cancer cells. **(A)** Immunofluorescence staining of THSD7B (red) and phosphorylated FAK [p-FAK(Y397), green] in AGS cells under NC, si-THSD7B, and THSD7B-overexpression conditions. Nuclei were counterstained with DAPI (blue). Representative merged images are shown. Scale bar = 50 μm. **(B)** Quantification of relative THSD7B fluorescence intensity. **(C)** Quantification of relative p-FAK(Y397) fluorescence intensity. Data are presented as mean ± SD. *P < 0.05, **P < 0.01 versus NC group.

To further visualize the association between THSD7B expression and FAK-related signaling activity, immunofluorescence staining was performed in AGS cells under THSD7B knockdown and overexpression conditions. As shown in Fig 7A, THSD7B immunofluorescence intensity was reduced in si-THSD7B cells and increased in THSD7B-overexpressing cells, consistent with the transfection efficiency. In parallel, phosphorylated FAK [p-FAK(Y397)] staining intensity showed a similar trend, with decreased fluorescence signals observed after THSD7B silencing and enhanced signals detected following THSD7B overexpression. Quantitative fluorescence analysis further supported these observations (Fig 7B and C).

Public protein annotation resources predict THSD7B as a membrane-associated protein and suggest potential involvement in actin cytoskeleton organization, although available subcellular annotation is not entirely uniform (https://www.ncbi.nlm.nih.gov/gene/80731). Therefore, the immunofluorescence results in the present study provide additional visual evidence that THSD7B expression is associated with FAK-related signaling activity in gastric cancer cells, but they do not establish a direct molecular interaction between THSD7B and FAK/SRC components.

### Restoration of FAK/SRC-associated signaling partially reverses changes induced by THSD7B silencing

**Fig 8:**
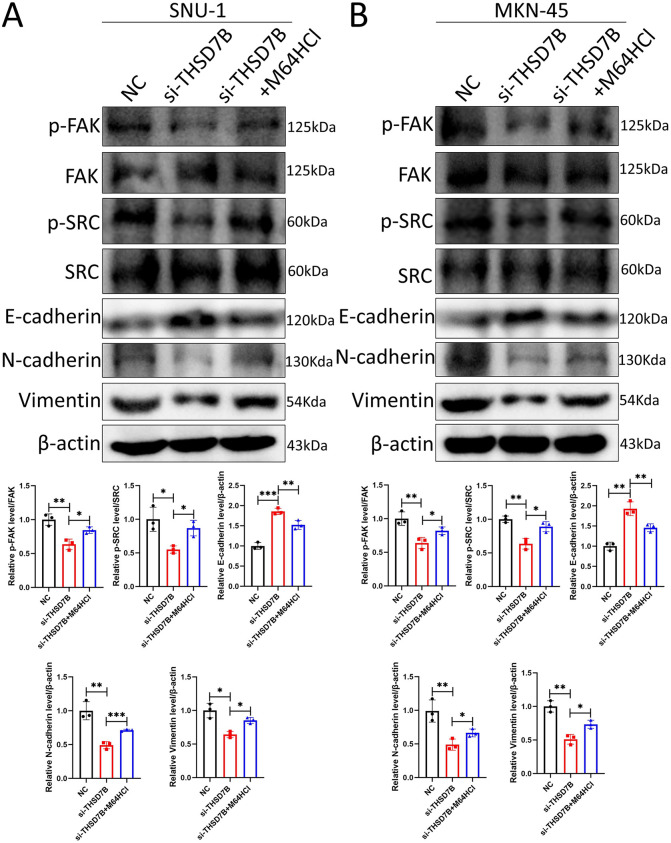
Rescue of signaling-related protein alterations by M64HCI treatment in THSD7B-silenced gastric cancer cells. **(A–B)** Western blot analysis of p-FAK, FAK, p-SRC, SRC, E-cadherin, N-cadherin, and vimentin in AGS and MKN-45 cells under three conditions: negative control (NC), THSD7B knockdown (si-THSD7B), and THSD7B knockdown with M64HCI treatment (si-THSD7B + M64HCI). β-actin was used as a loading control. Relative protein expression levels were quantified by densitometric analysis and normalized to β-actin or corresponding total protein levels. Data are presented as mean ± SD. *P < 0.05, **P < 0.01, ***P < 0.001.

To further examine whether the observed signaling alterations were related to FAK/SRC activity, a rescue experiment was performed using the FAK pathway activator M64HCI in THSD7B-silenced gastric cancer cells. Western blot analysis showed that THSD7B knockdown was associated with reduced levels of phosphorylated FAK (p-FAK) and phosphorylated SRC (p-SRC), while total FAK and SRC expression remained relatively unchanged. Upon treatment with M64HCI, the levels of p-FAK and p-SRC were partially restored in both AGS and MKN-45 cells. In parallel, changes in epithelial–mesenchymal phenotype–related proteins were also examined. THSD7B silencing was accompanied by increased E-cadherin expression and decreased N-cadherin and vimentin levels. Following M64HCI treatment, these alterations showed a tendency toward reversal, with reductions in E-cadherin and increases in N-cadherin and vimentin expression compared with the THSD7B knockdown group (Fig 8A and B). These findings indicate that modulation of FAK/SRC-associated signaling may be involved in the molecular changes observed after THSD7B knockdown.

### Schematic model of THSD7B-associated signaling in gastric adenocarcinoma

**Fig 9:**
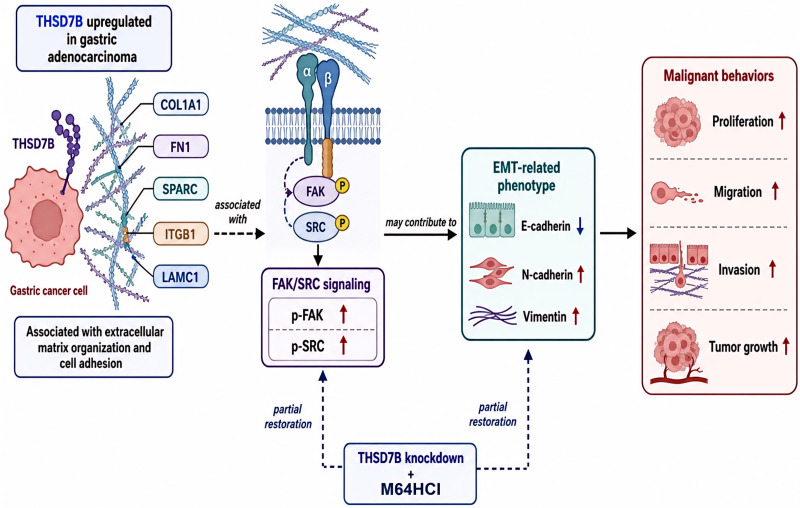
Proposed model of THSD7B-associated signaling in gastric adenocarcinoma. THSD7B is upregulated in gastric adenocarcinoma and is associated with extracellular matrix organization and cell adhesion-related processes. THSD7B expression correlates with ECM-associated genes, including COL1A1, FN1, SPARC, ITGB1, and LAMC1, suggesting a potential link with adhesion-related signaling. Altered THSD7B expression is accompanied by changes in FAK/SRC signaling activity, characterized by increased p-FAK and p-SRC levels. These signaling changes may contribute to EMT-related phenotypic alterations, including decreased E-cadherin and increased N-cadherin and Vimentin expression. Functionally, this process is associated with enhanced proliferation, migration, invasion, and tumor growth. M64HCI partially restores signaling and EMT-related changes induced by THSD7B knockdown, supporting the involvement of FAK/SRC-associated signaling in this process. This schematic represents a proposed model based on the findings of the present study and does not imply a confirmed direct molecular interaction.

To provide an integrated interpretation of the bioinformatic and experimental findings, we summarized the potential role of THSD7B in gastric adenocarcinoma in a schematic model (Fig 9). In this model, THSD7B upregulation is linked to extracellular matrix organization and cell adhesion-related biological processes, consistent with the enrichment results and the observed correlations between THSD7B and ECM-associated genes, including COL1A1, FN1, SPARC, ITGB1, and LAMC1. These adhesion-related changes may be associated with activation of FAK/SRC signaling, as reflected by increased phosphorylation of FAK and SRC in THSD7B-overexpressing cells and reduced phosphorylation after THSD7B knockdown. Downstream of this signaling context, THSD7B modulation was accompanied by changes in EMT-related markers, including decreased E-cadherin and increased N-cadherin and Vimentin, which may contribute to enhanced proliferation, migration, invasion, and tumor growth. In addition, the partial reversal observed after M64HCI treatment further supports the possibility that FAK/SRC-associated signaling participates in the biological effects related to THSD7B. Overall, this model suggests that THSD7B may contribute to gastric cancer progression through an adhesion-related signaling network, while the precise molecular interactions require further validation.

## Discussion

In this study, THSD7B was found to be upregulated in gastric cancer and associated with patient prognosis in public datasets. Multivariate Cox regression analysis further suggested that THSD7B retained prognostic relevance after adjustment for clinicopathological variables. And external validation supporting its differential expression. Functional experiments further showed that modulation of THSD7B affected proliferation, migration, and invasion of gastric cancer cells, suggesting that THSD7B is linked to malignant cellular phenotypes rather than representing a purely correlative finding.

Functional enrichment analyses consistently indicated involvement of adhesion-related processes and cytoskeleton-associated pathways. This observation is in line with previous studies showing that cell adhesion and related signaling contribute to tumor progression by influencing cellular motility and plasticity. Consistently, THSD7B expression was positively correlated with several adhesion-associated genes, including COL1A1, FN1, SPARC, ITGB1, and LAMC1 [15,16]. Although the strength of individual correlations was modest, these findings may reflect a broader association between THSD7B expression and adhesion-related transcriptional states in gastric adenocarcinoma.

At the signaling level, alterations in THSD7B expression were accompanied by changes in FAK/SRC-related signaling and EMT-associated markers. Rather than indicating a direct regulation of a specific pathway, these findings support the notion that THSD7B is associated with adhesion-related signaling states that are linked to tumor cell motility. This interpretation is consistent with the established role of FAK/SRC signaling in‌‌ regulating migration and invasion in cancer [17–19].

The clinical analyses in this study did not demonstrate a strictly monotonic relationship between THSD7B expression and all pathological subgroups, which may reflect the inherent heterogeneity of gastric adenocarcinoma. Therefore, THSD7B is more appropriately interpreted as a biomarker associated with tumor behavior and patient prognosis rather than a simple indicator of disease stage. This observation is consistent with the multifactorial nature of tumor progression, where molecular alterations do not always parallel conventional clinicopathological parameters.

The functional findings were supported by both in vitro experiments and in vivo tumor growth assays, suggesting that the observed effects are not limited to a single experimental setting. However, several limitations should be acknowledged. The study partially relies on retrospective public datasets, and although experimental validation was performed, the underlying molecular mechanisms were not fully explored. In addition, further studies are required to clarify the potential clinical applicability of THSD7B in larger patient cohorts. Moreover, Although knockdown-based xenograft experiments supported the in vivo relevance of THSD7B, overexpression-based animal experiments were not included, which represents a limitation of the present study and should be addressed in future work.

In summary, THSD7B is associated with tumor progression and patient outcomes in gastric adenocarcinoma. The present findings suggest that THSD7B may have potential relevance as a biomarker candidate and could contribute to the understanding of tumor behavior, while further investigation ‌‌is needed to confirm its clinical utility and underlying biological significance.

## Supporting information

S1 Data Raw Data. (XLSX)

S1 File Original western blot. (PDF)
