# Supplementary material for: THSD7B promotes tumor progression and is associated with prognosis in gastric adenocarcinoma
Source: PLoS One. 2026 Jun 12;21(6):e0351545. doi: 10.1371/journal.pone.0351545 (PMC13262811; doi:10.1371/journal.pone.0351545)

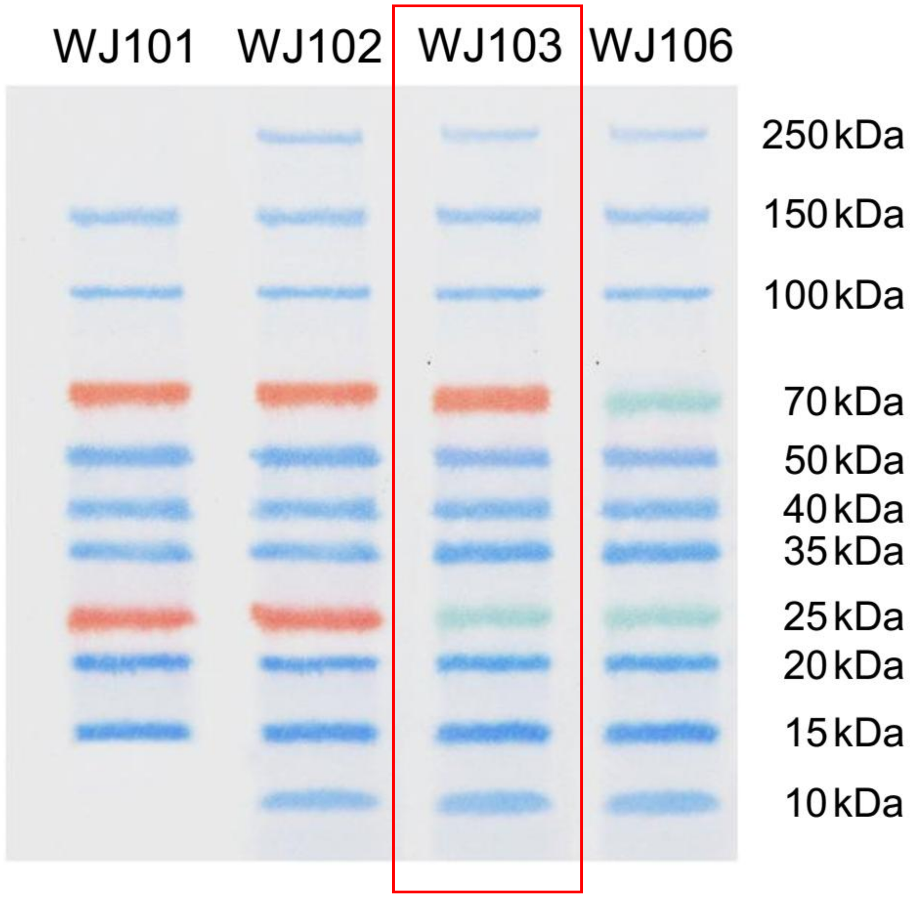

Figure 6:

THSD7B-1：

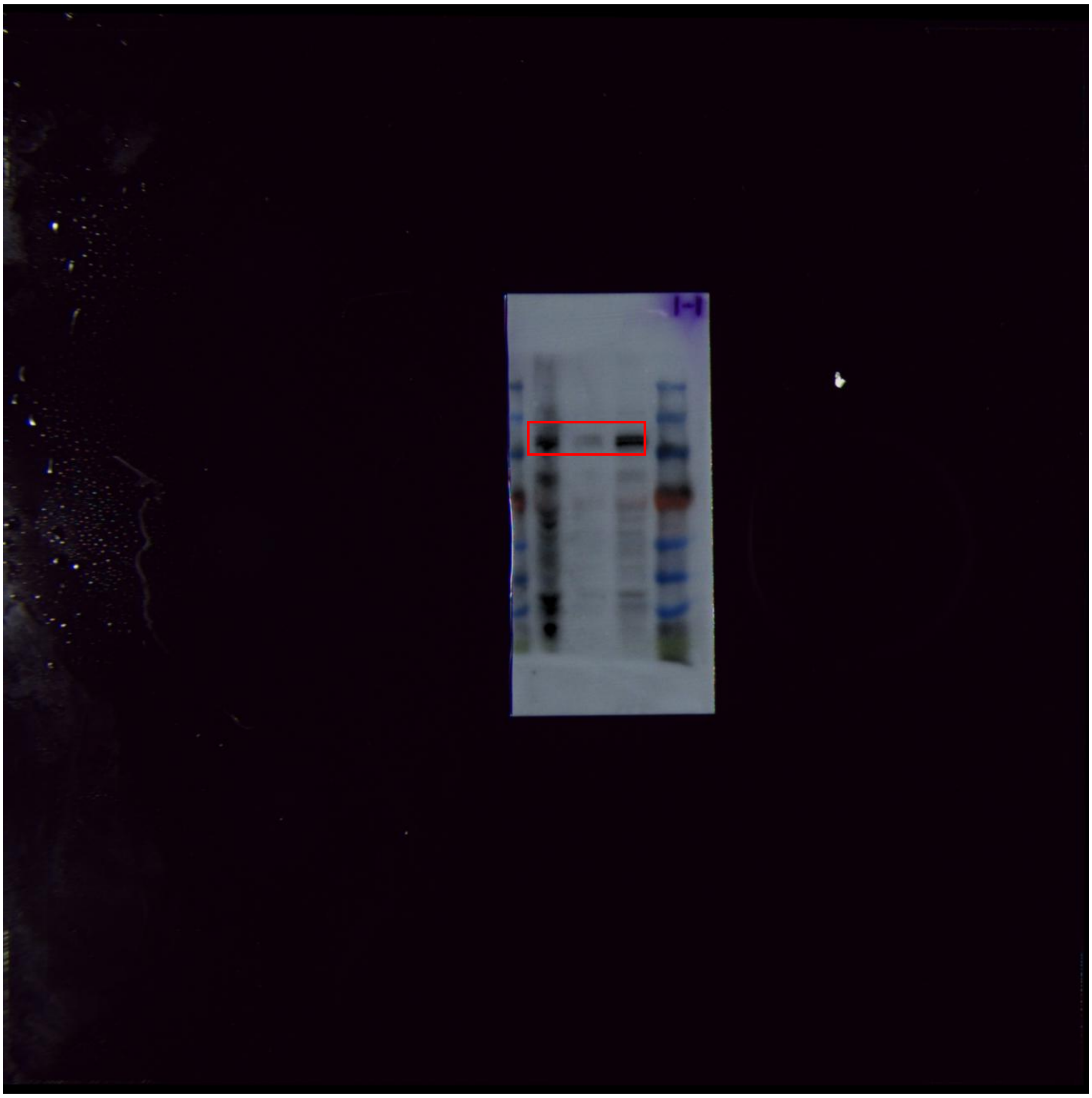

THSD7B-2：

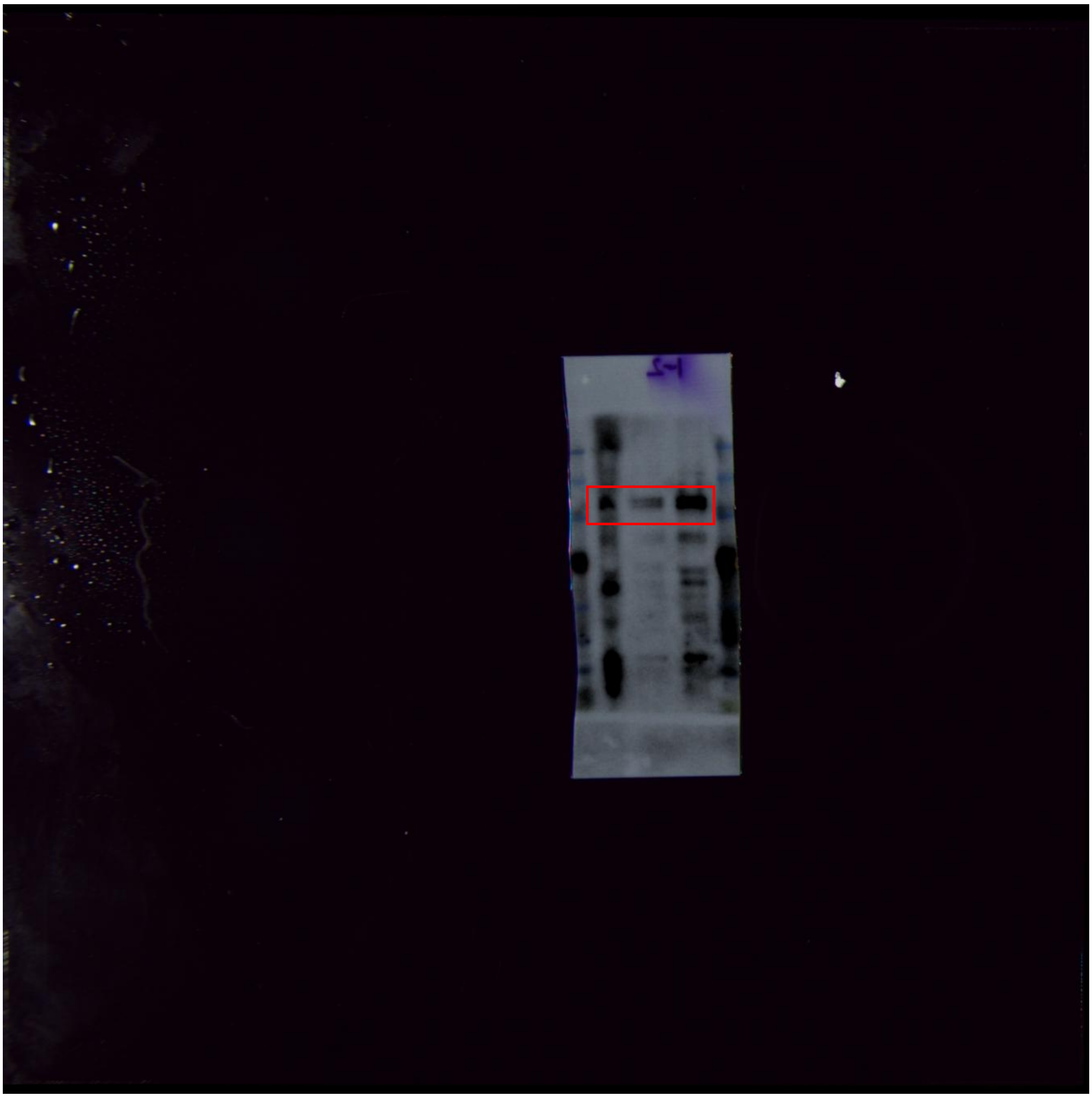

P-FAK-1:

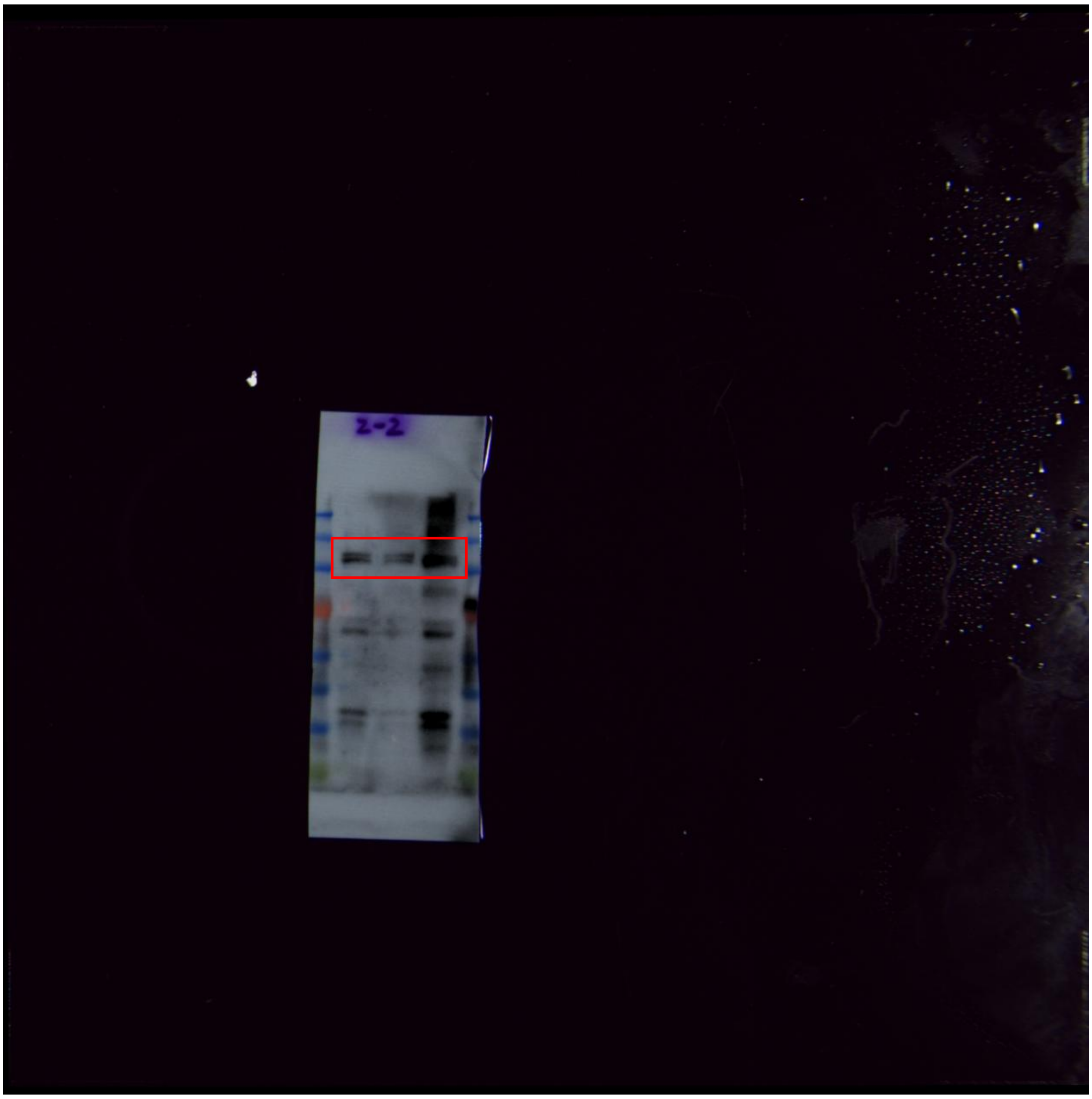

p-FAK-2:

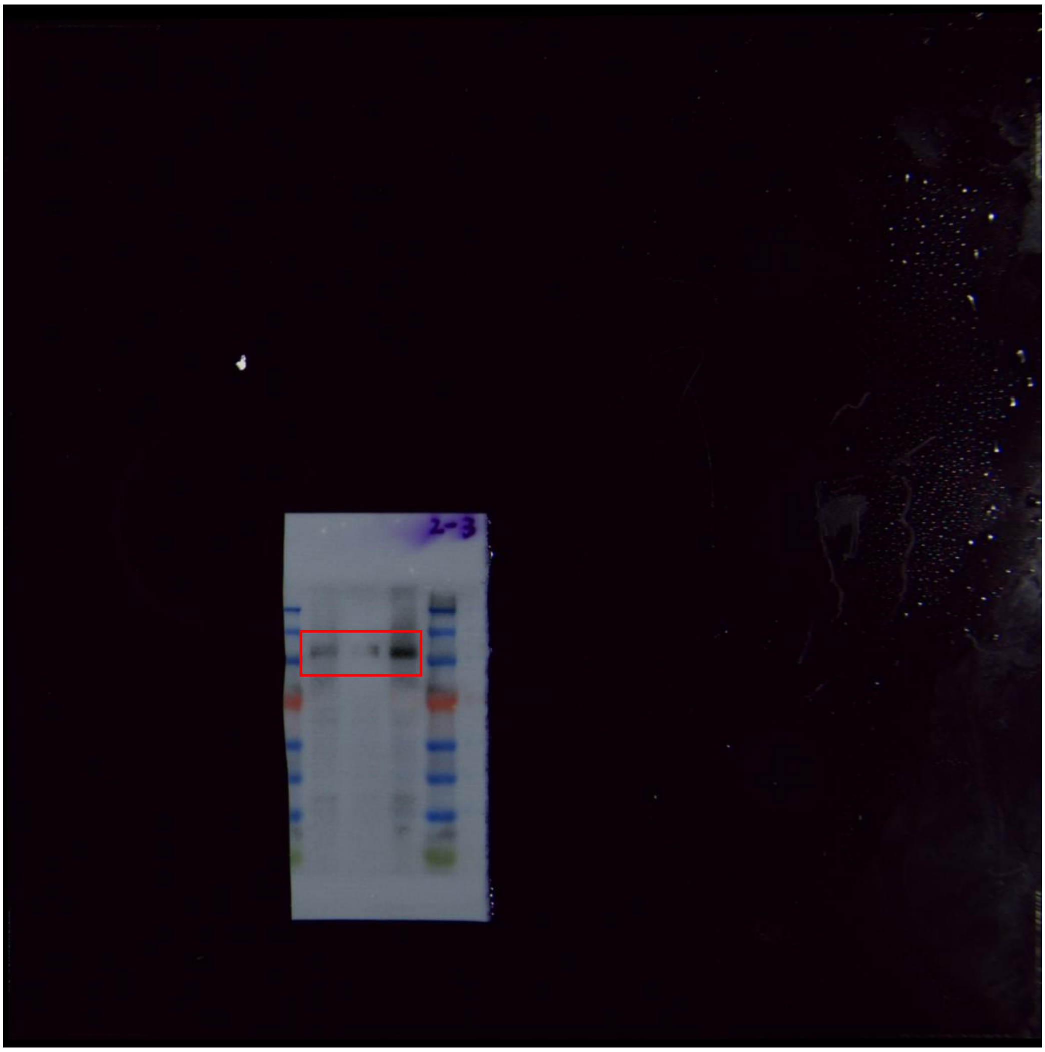

FAK-1:

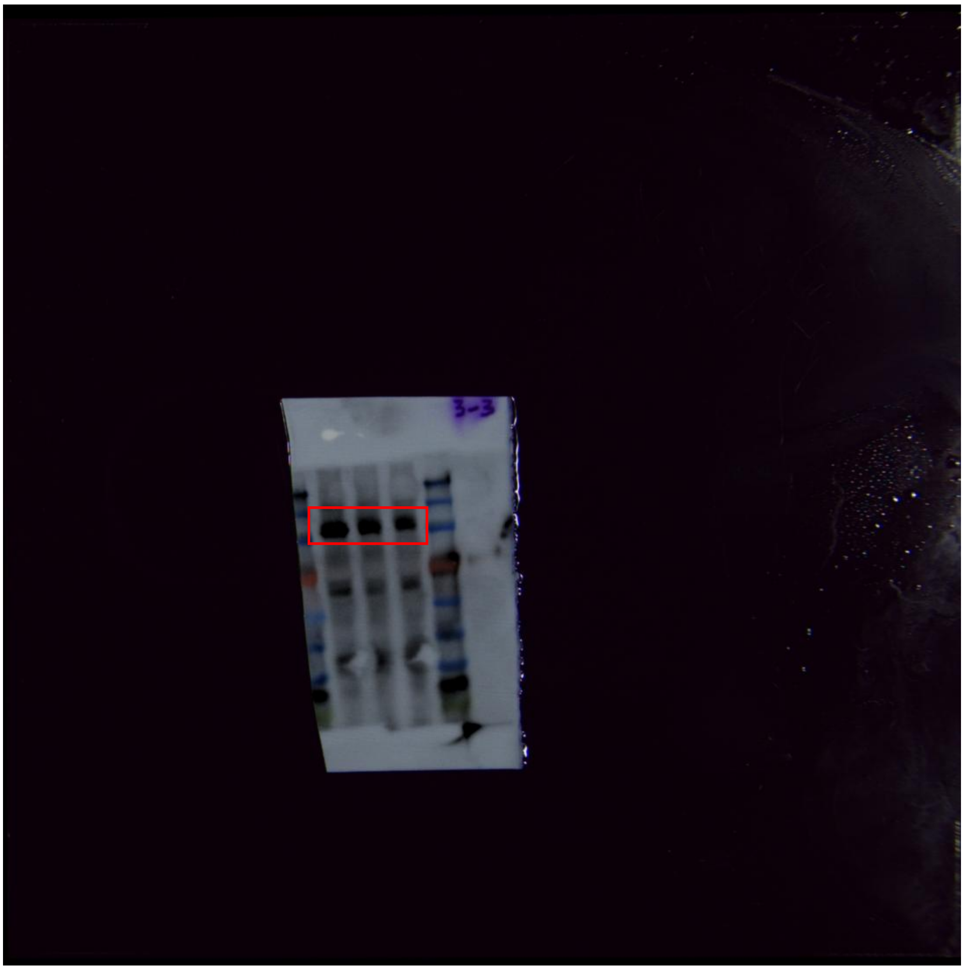

FAK-2:

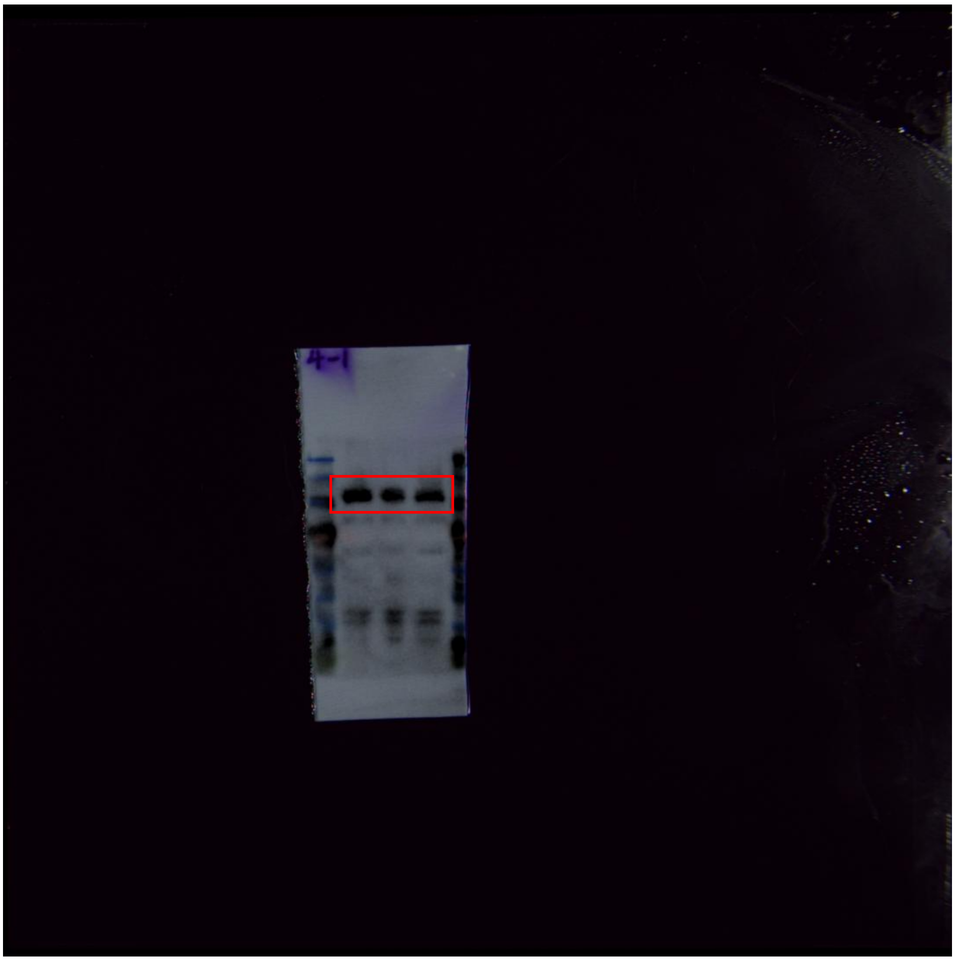

P-SRC-1:

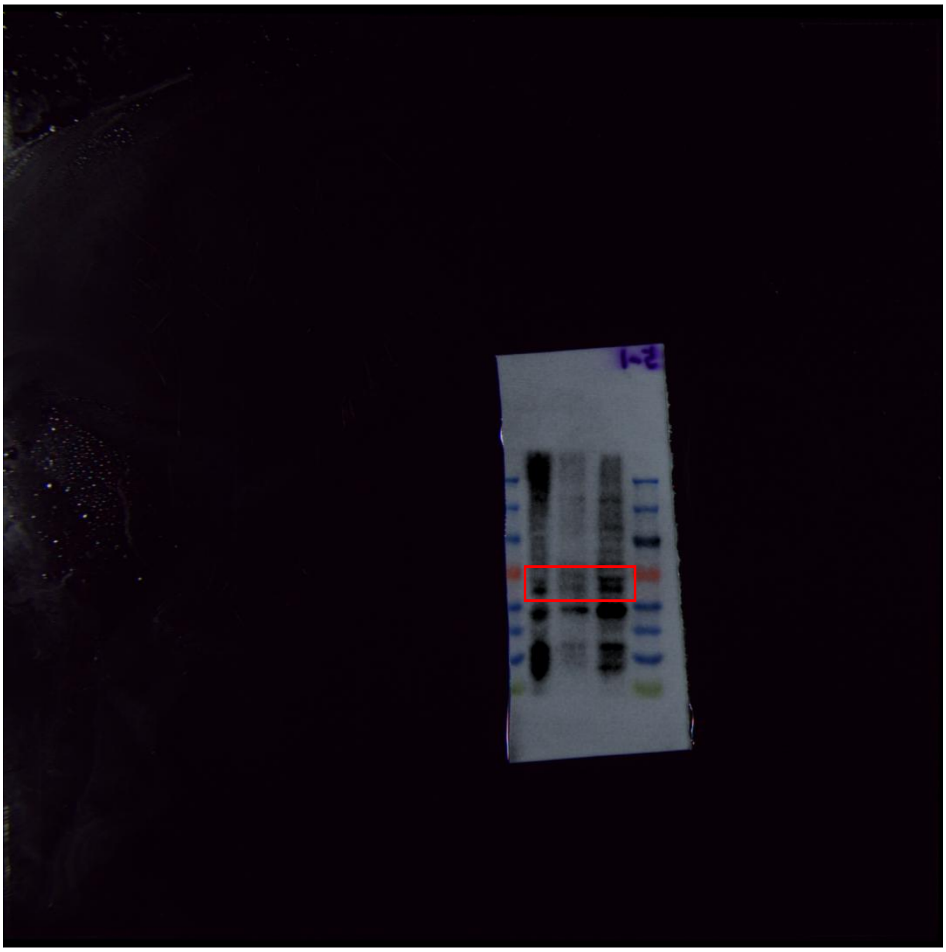

P-SRC-2:

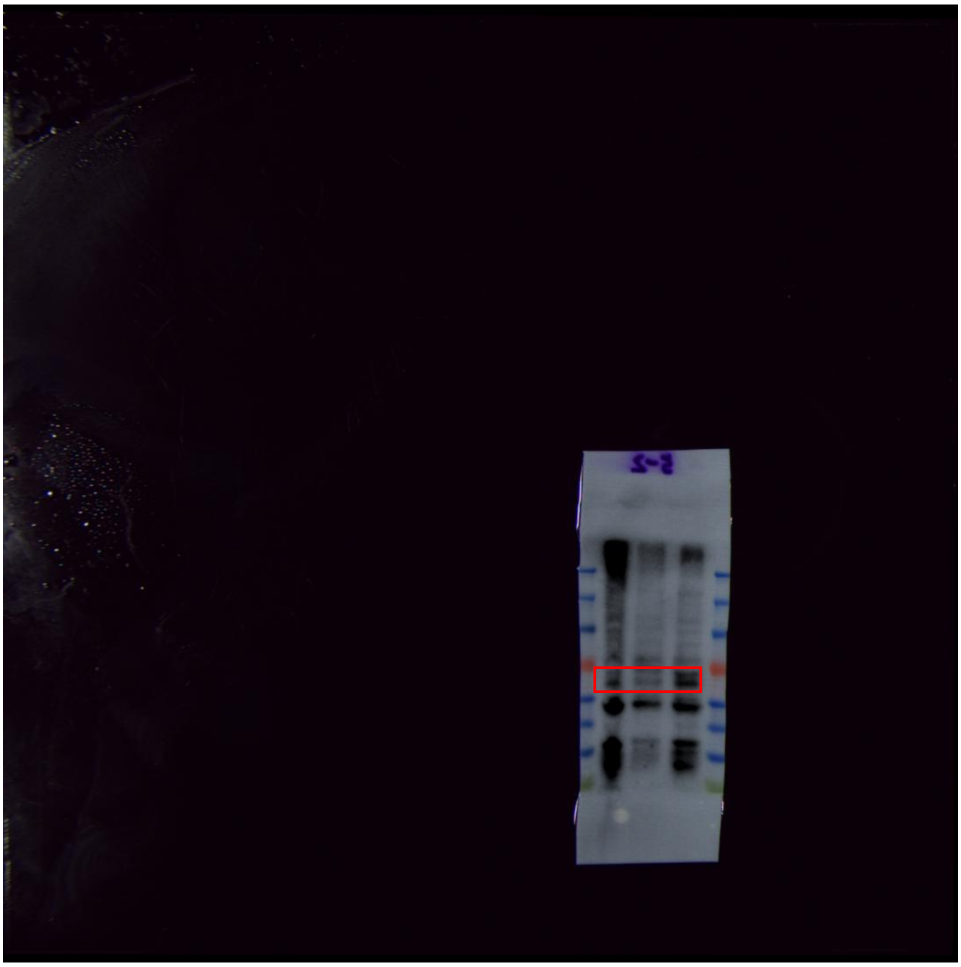

SRC-1:

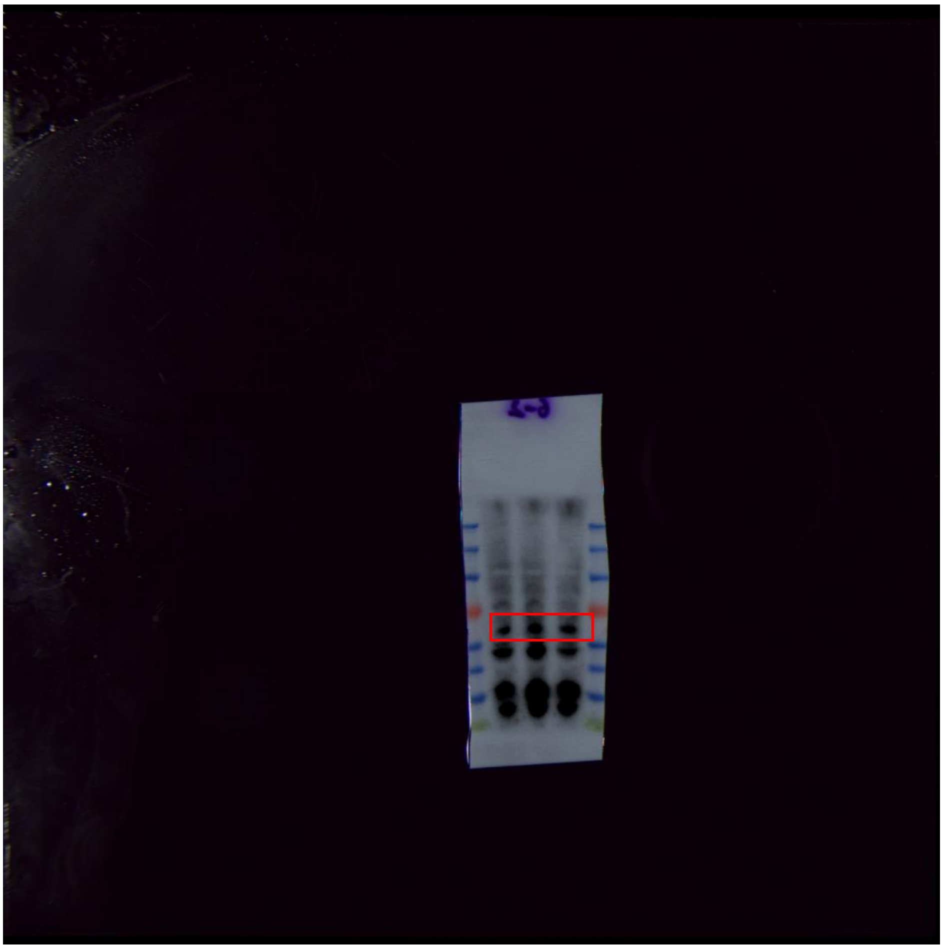

SRC-2:

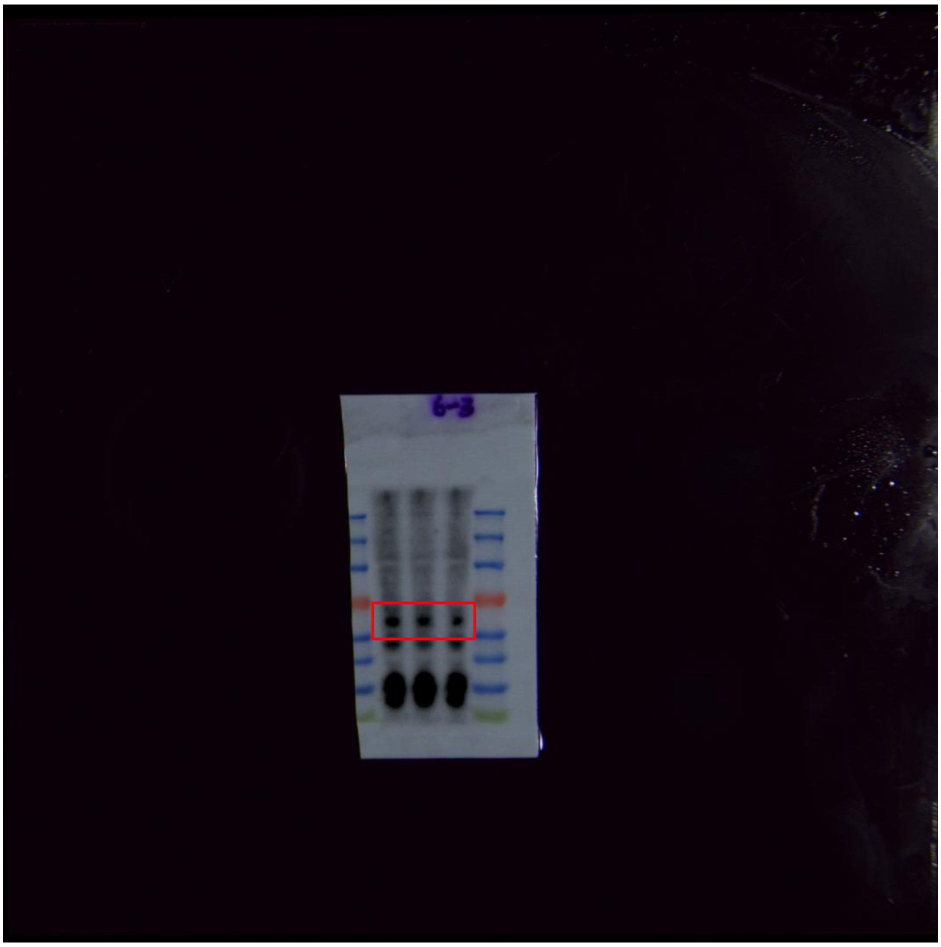

E-cadherin-1:

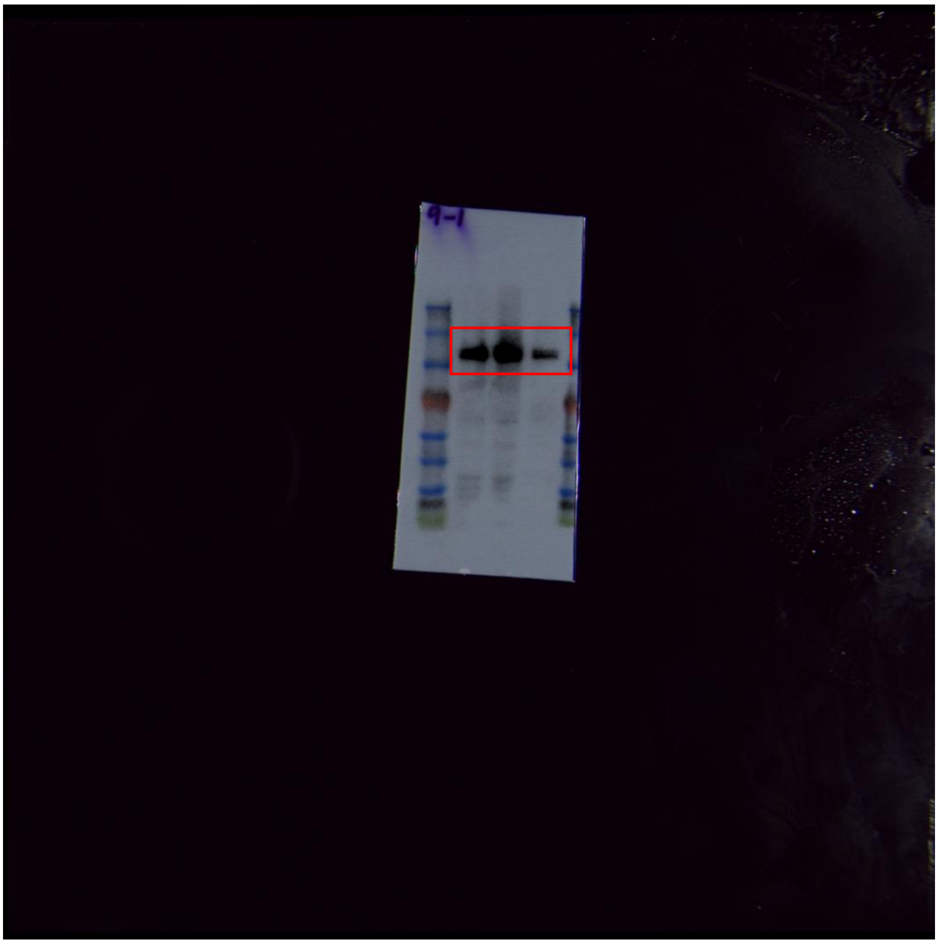

E-cadherin-2:

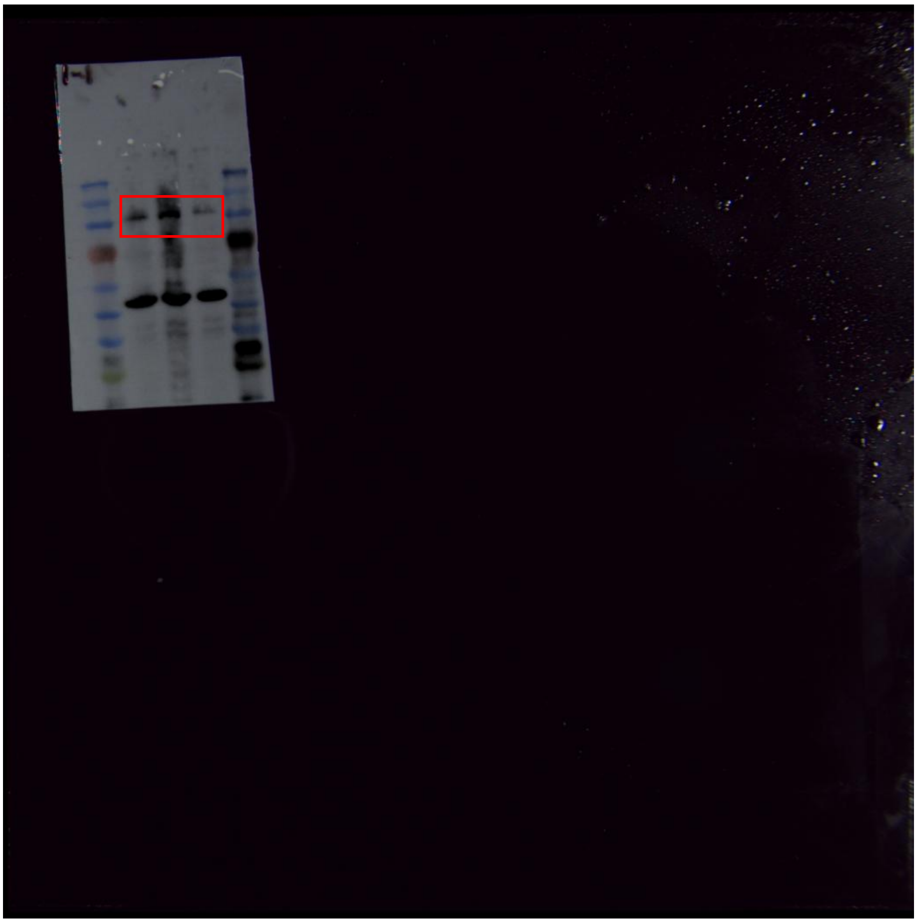

N-cadherin-1:

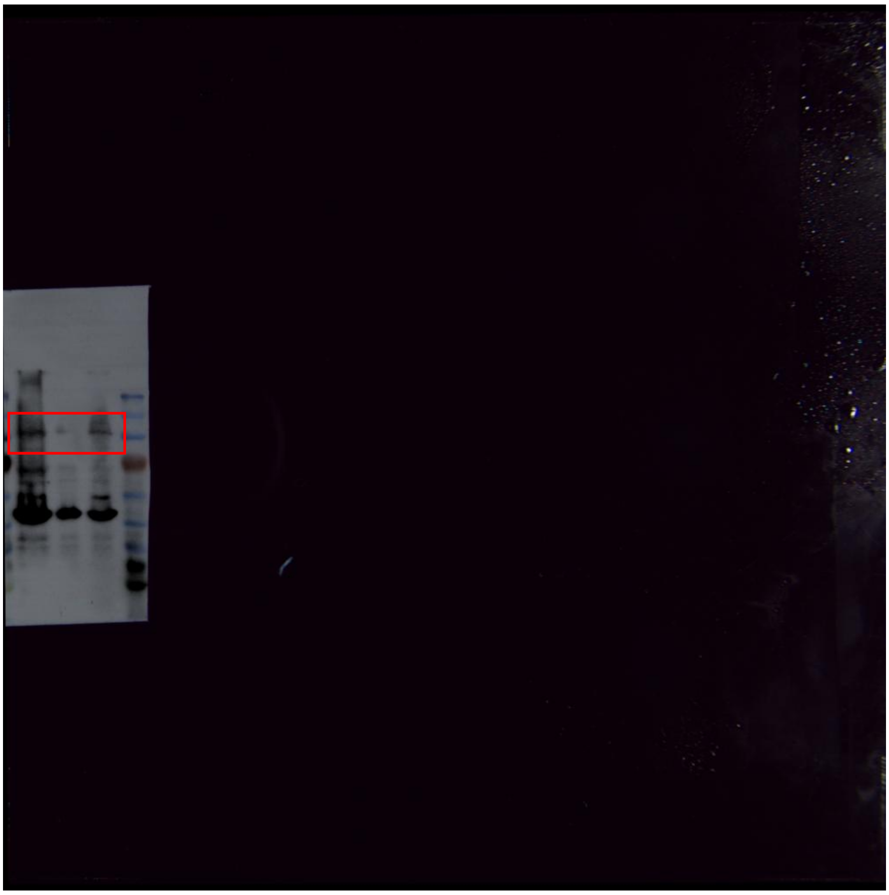

N-cadherin-2:

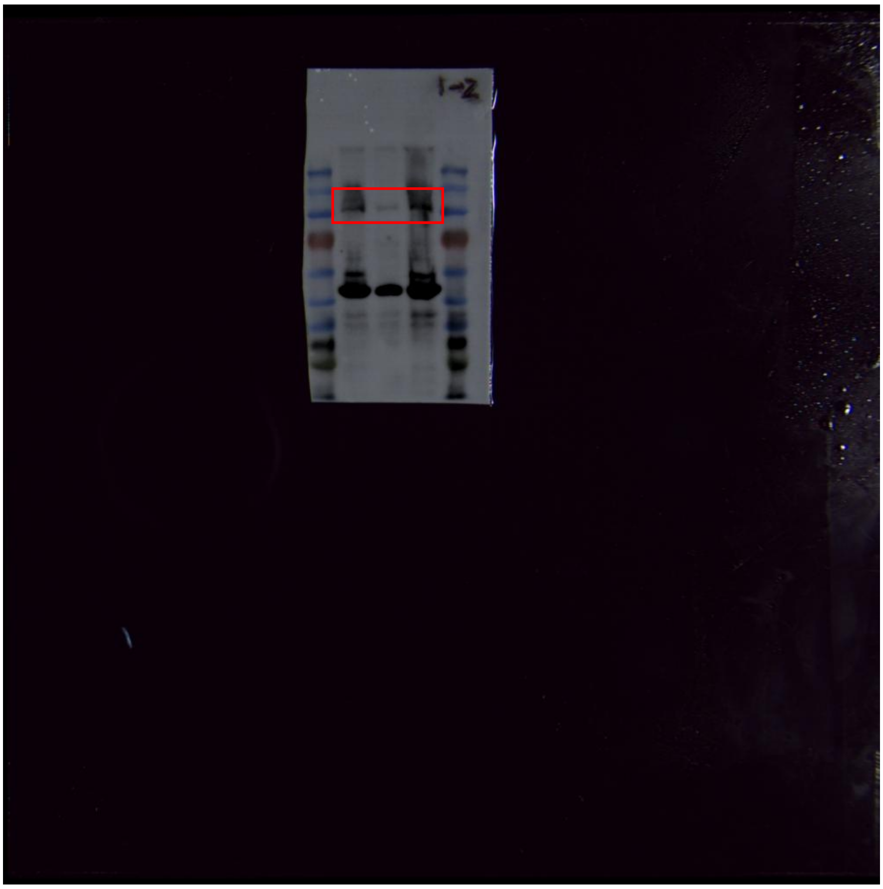

Vimentin-1:

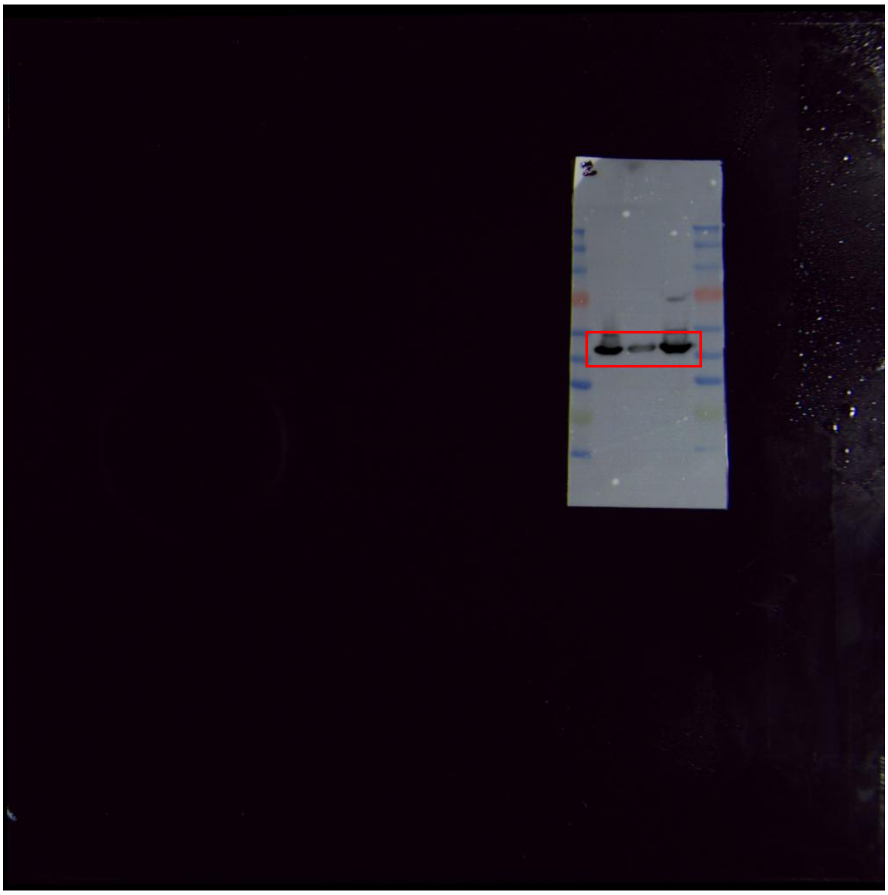

Vimentin-2:

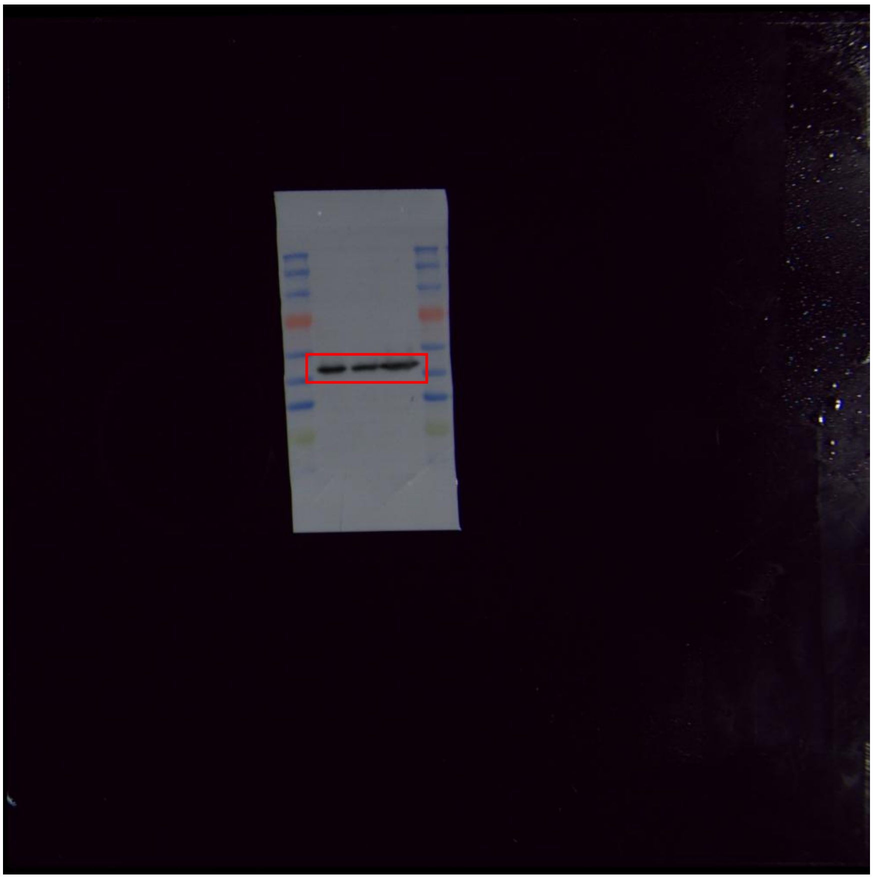

Β -actin-1:

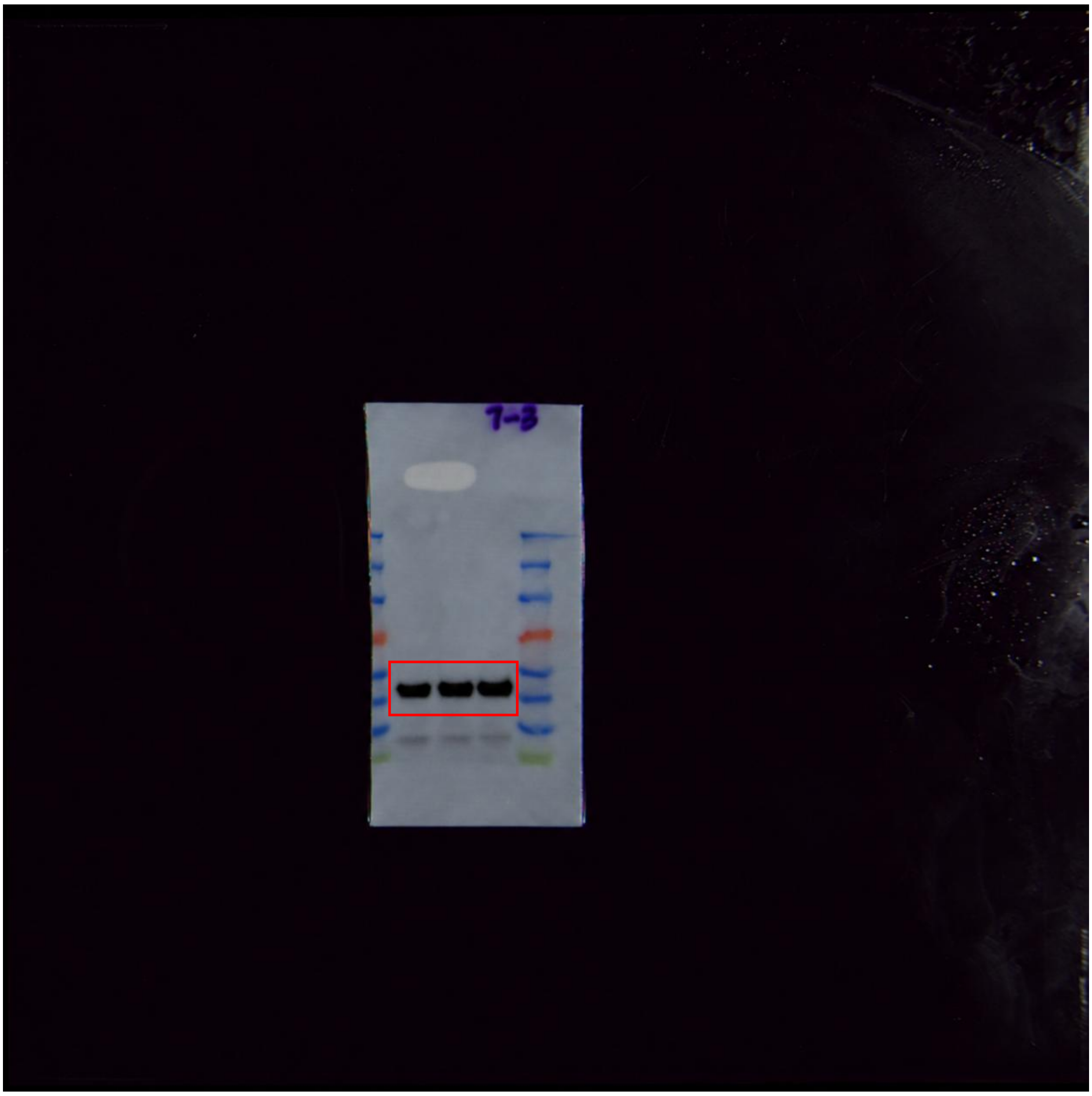

Β -actin-2：

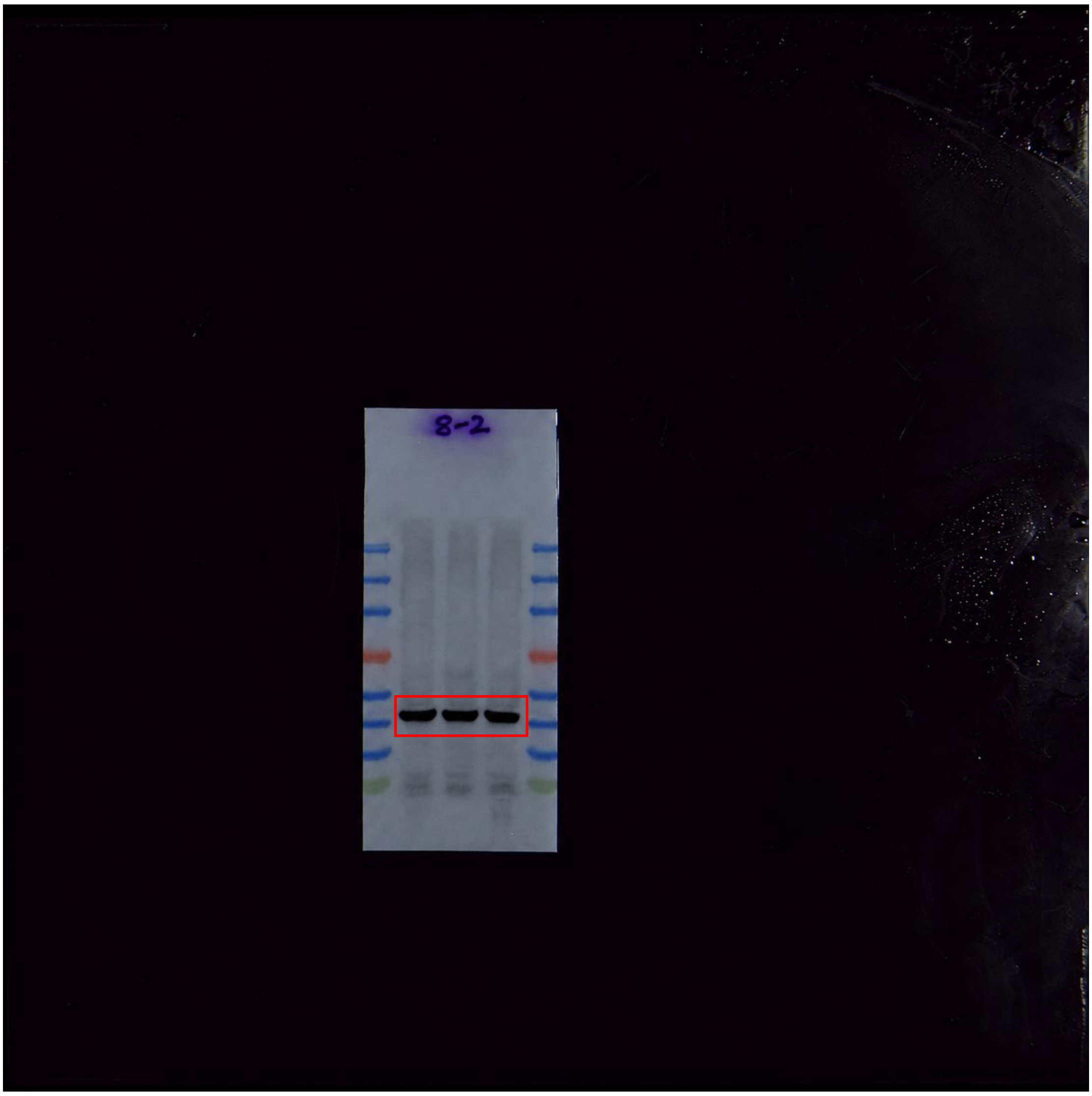
8-2

Figure 8:

P-FAK-1:

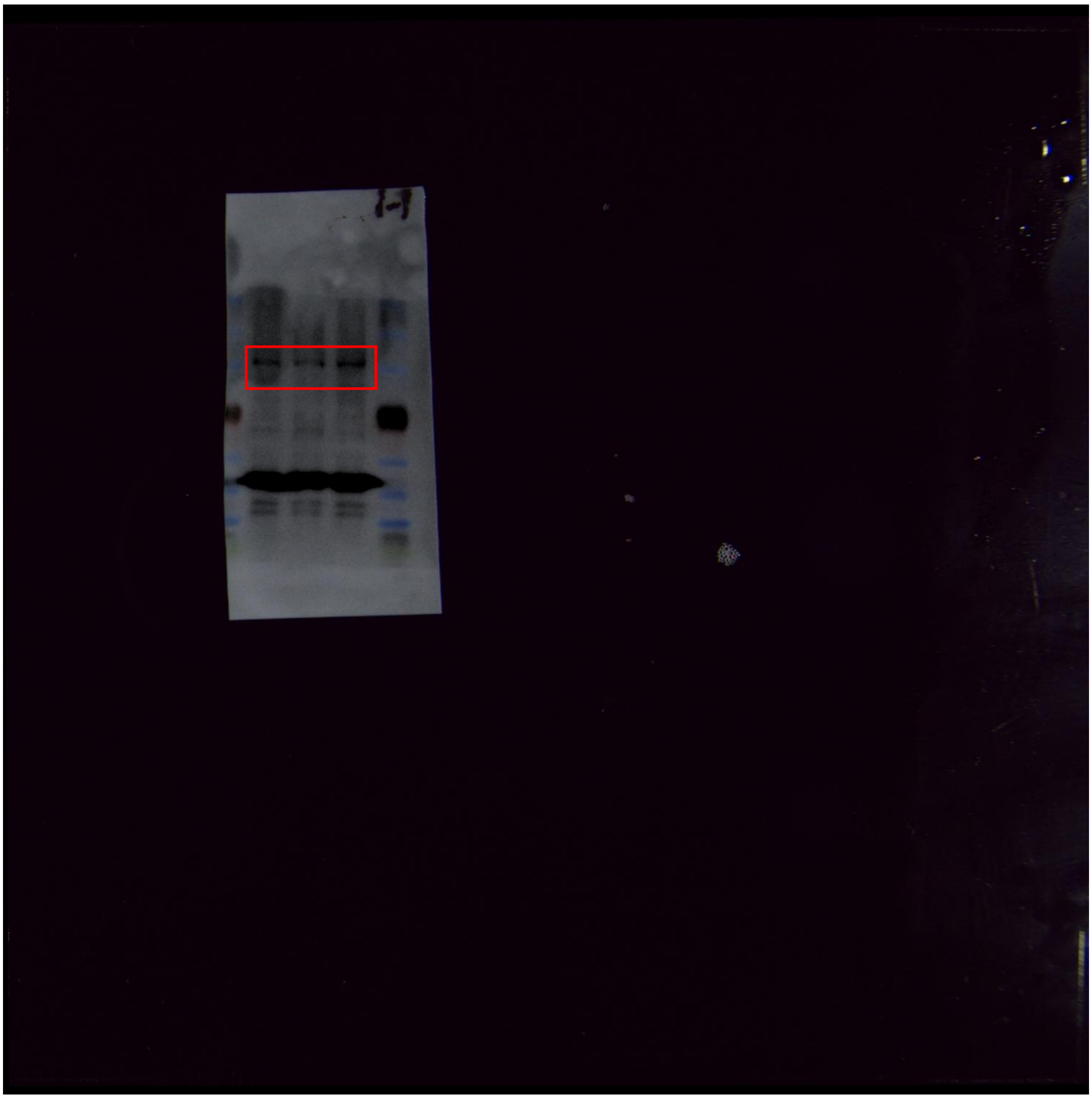

P-FAK-2:

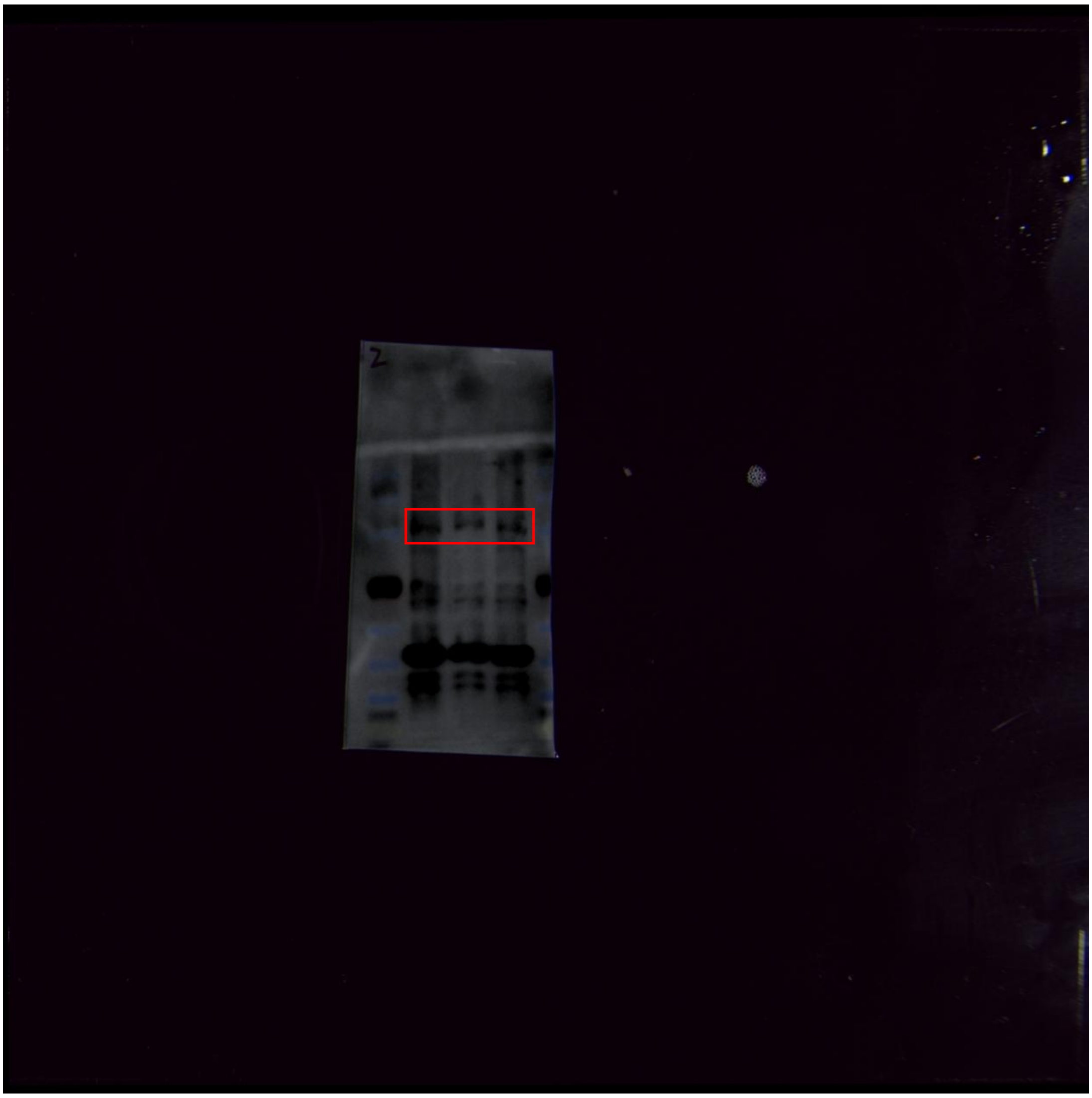

FAK-1:

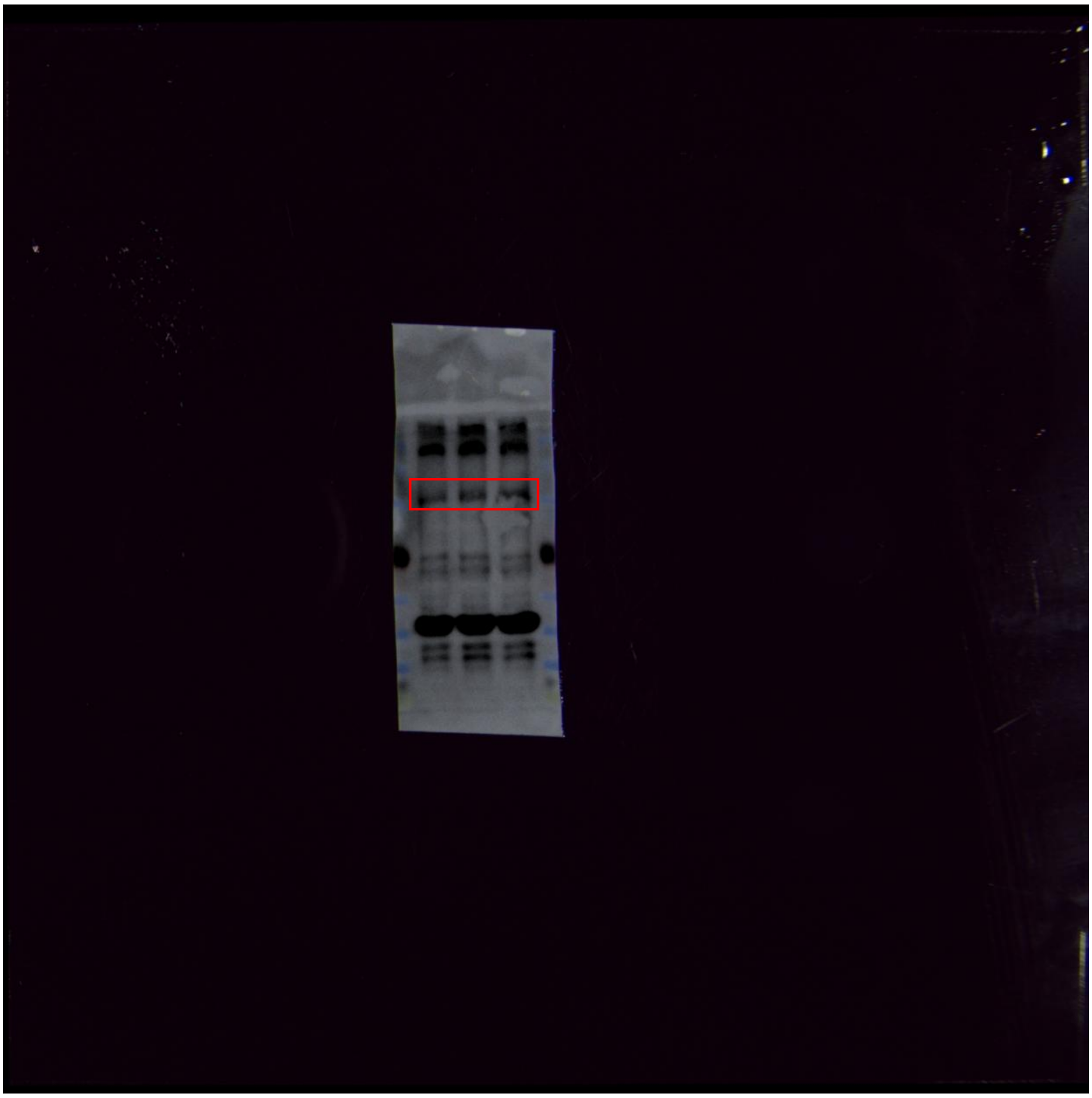

FAK-2:

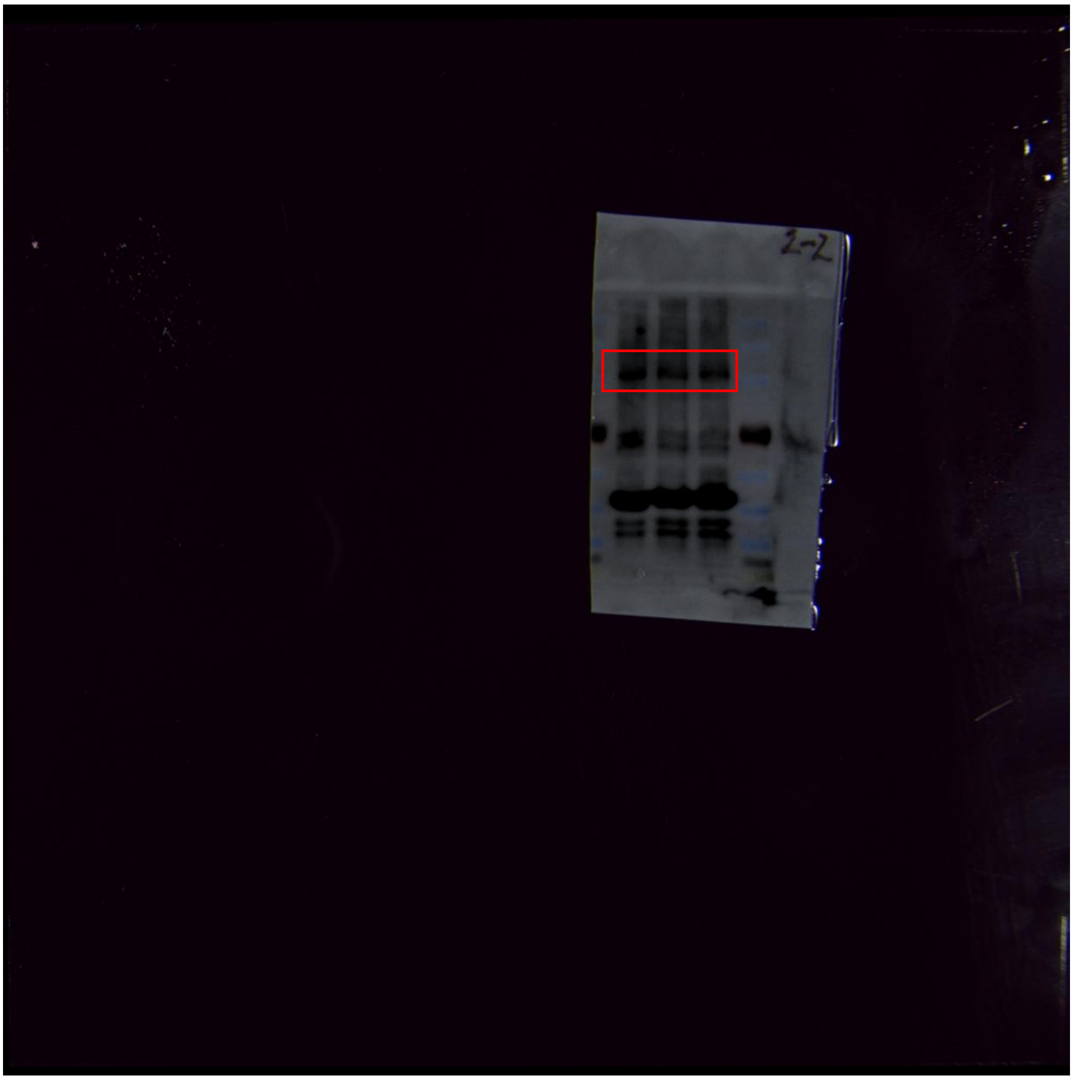

P-SRC-1:

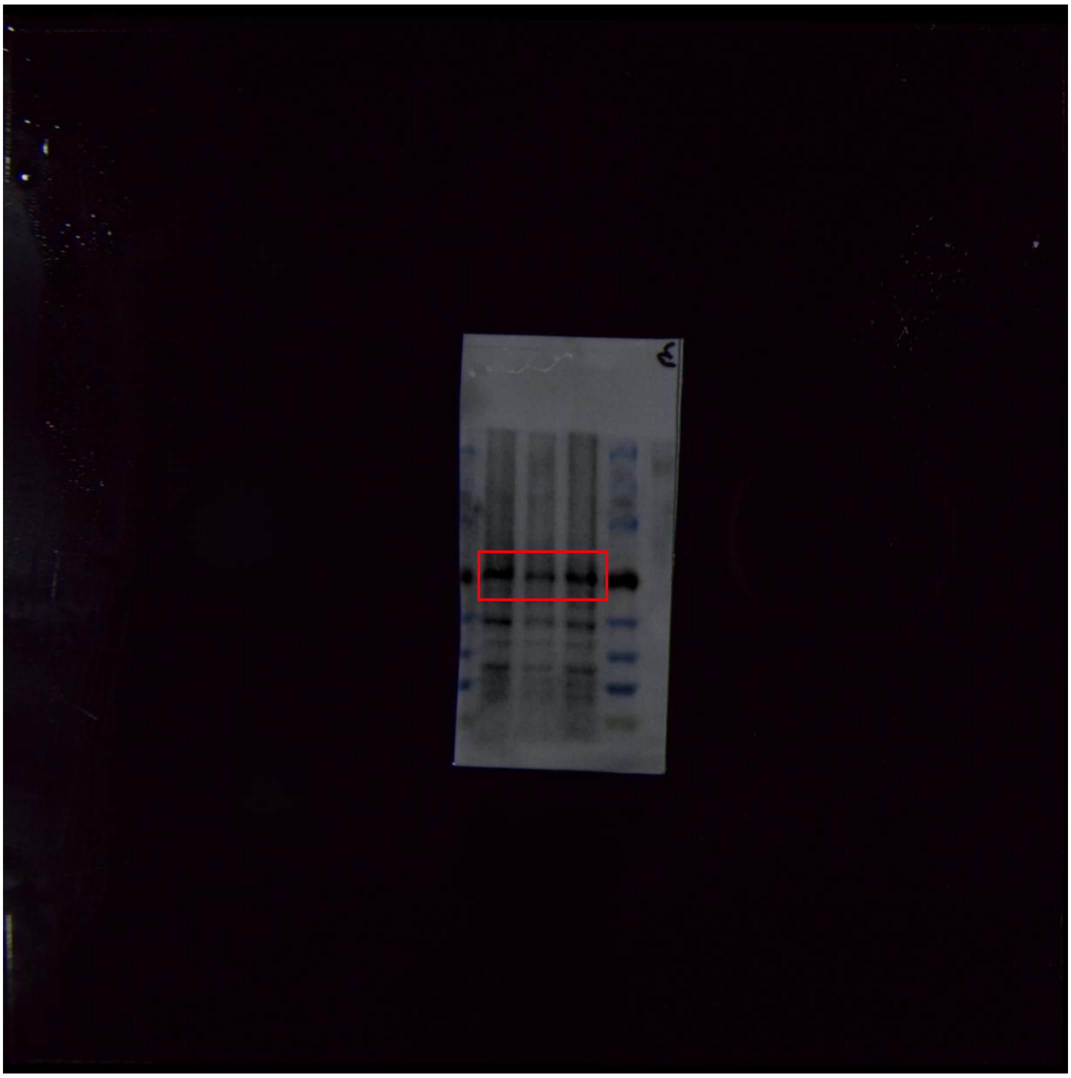

P-SRC-2:

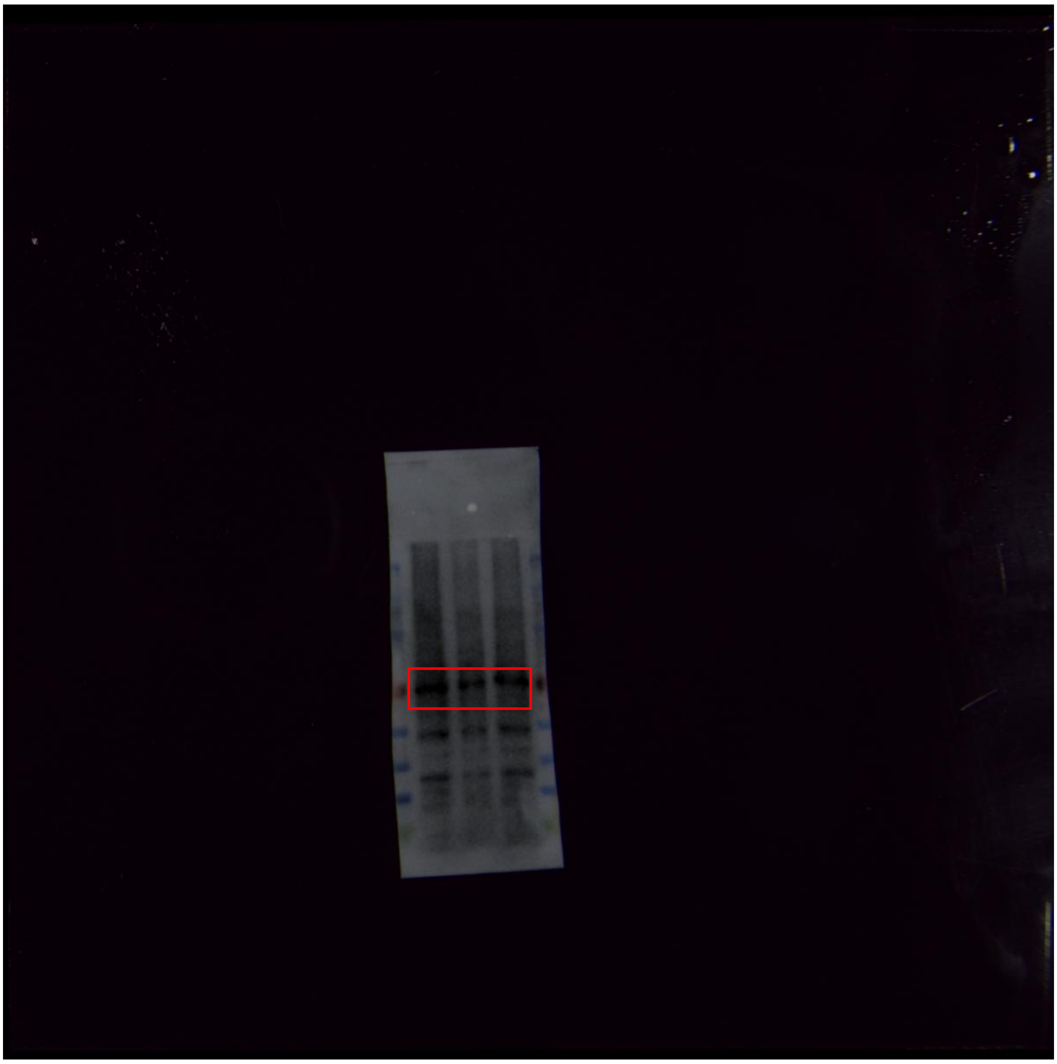

SRC-1：

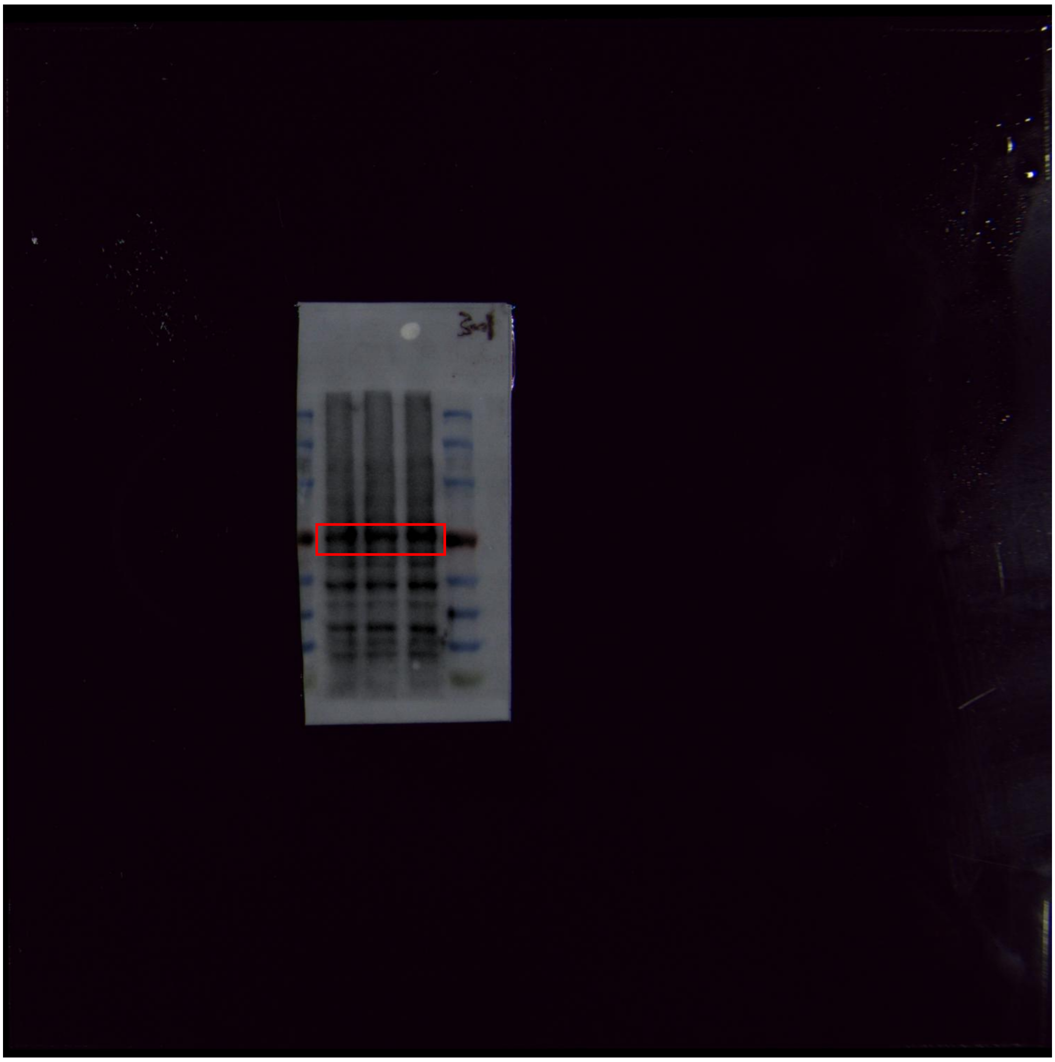

SRC-2：

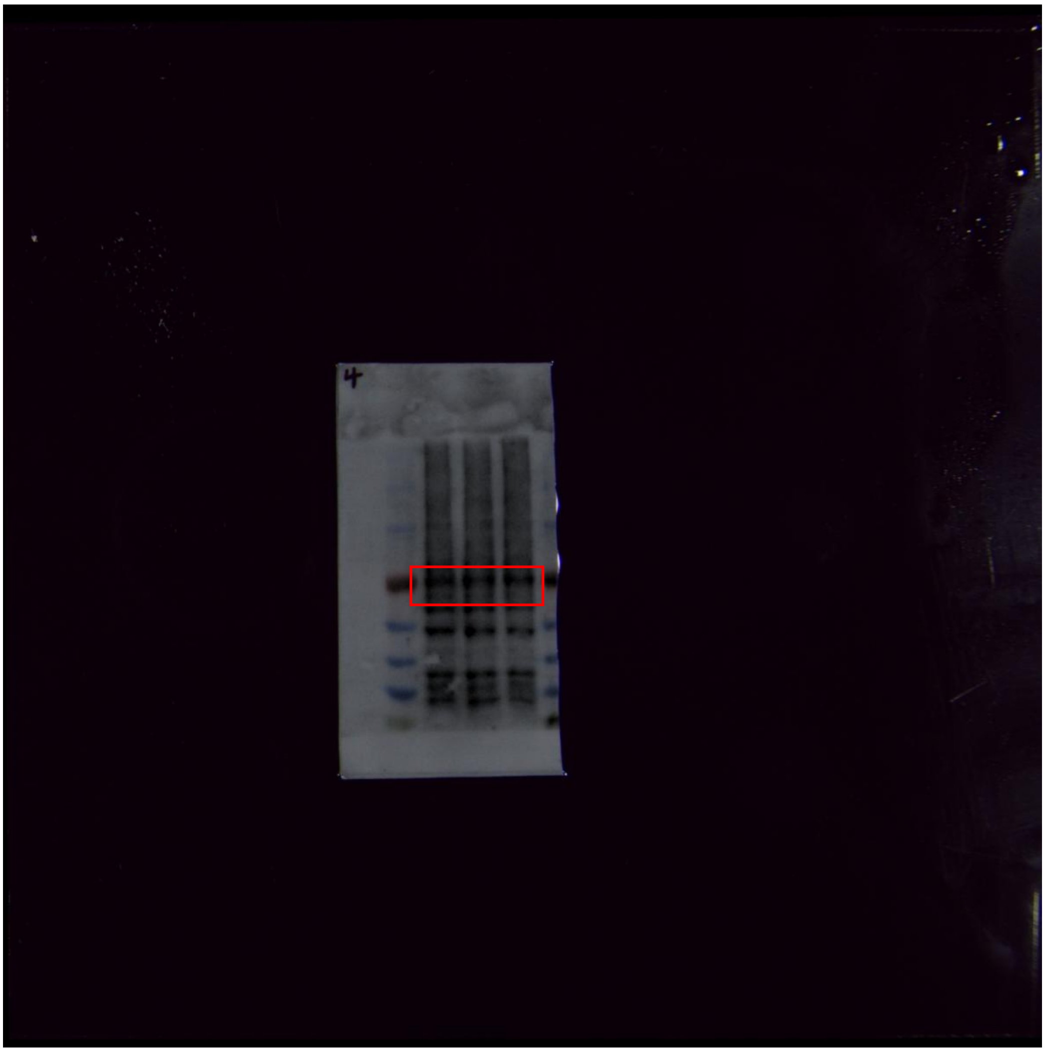

E-cadherin-1:

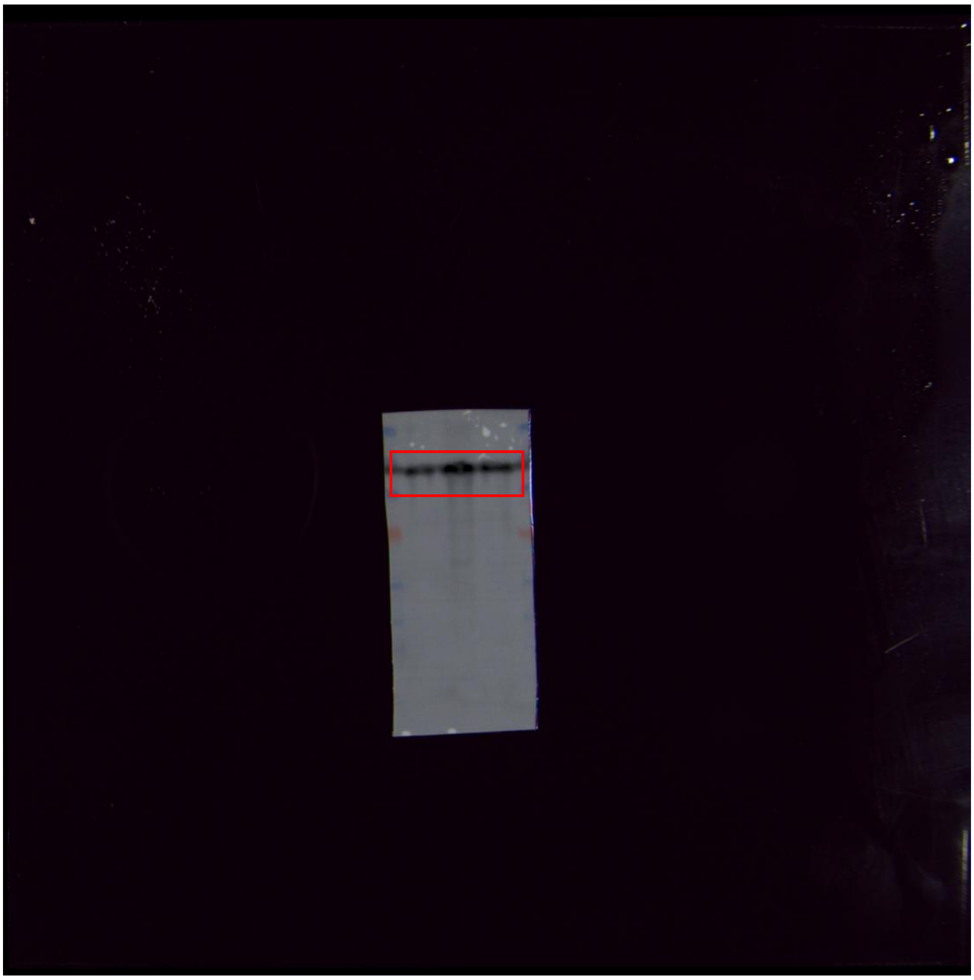

E-cadherin-2:

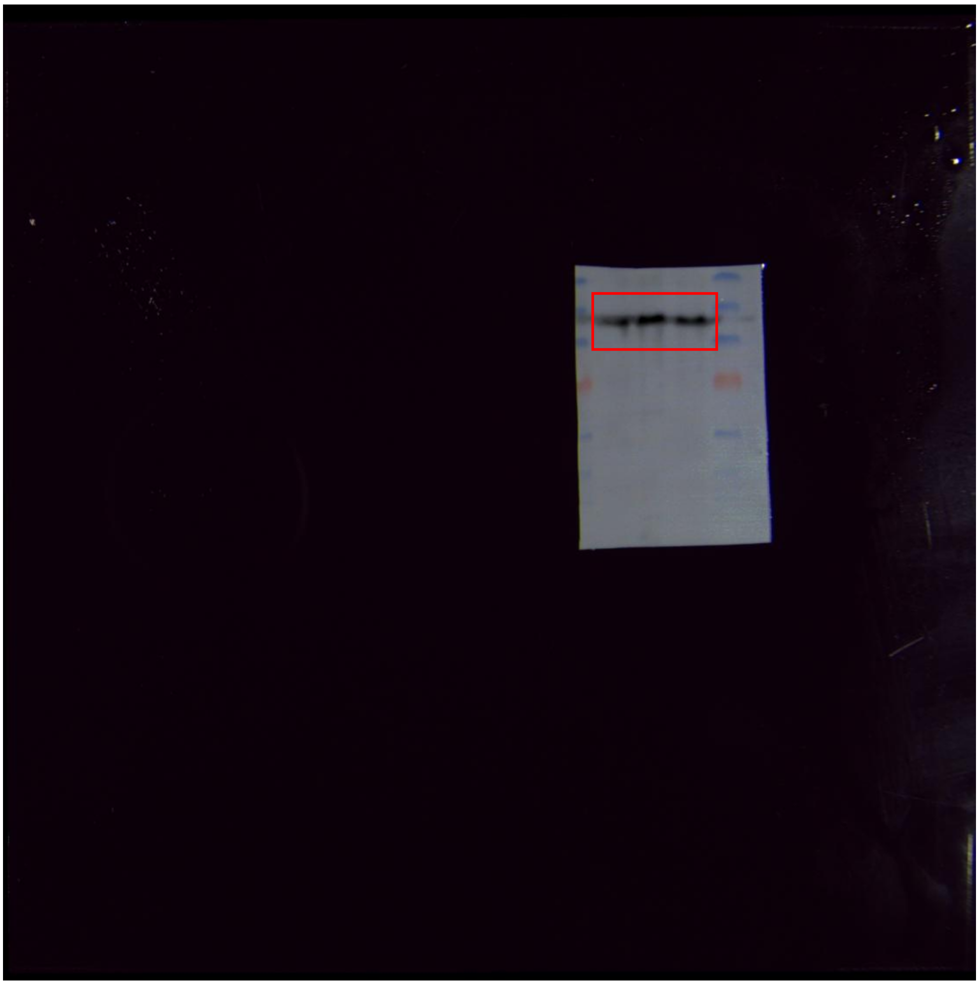

N-cadherin-1:

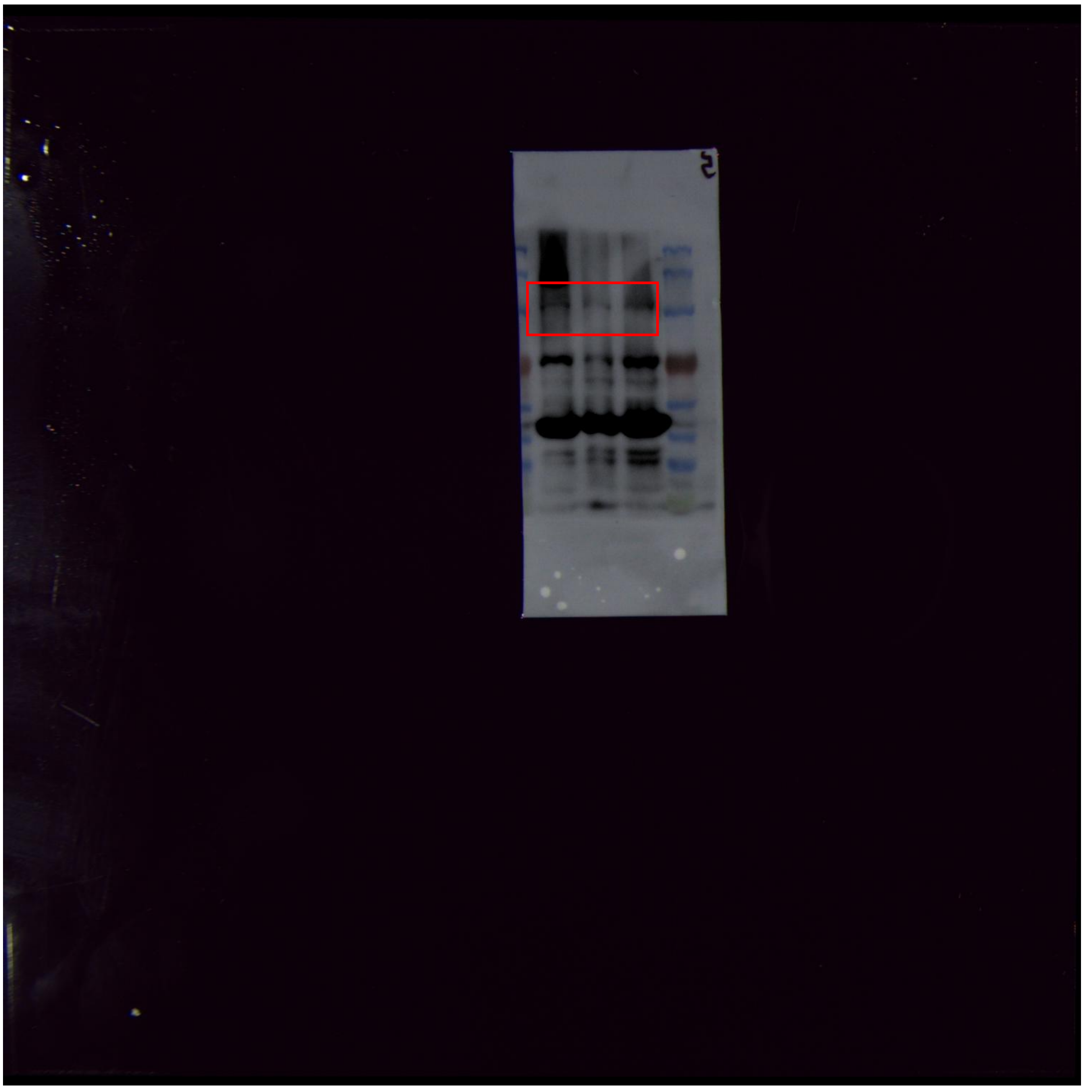

N-cadherin-2:

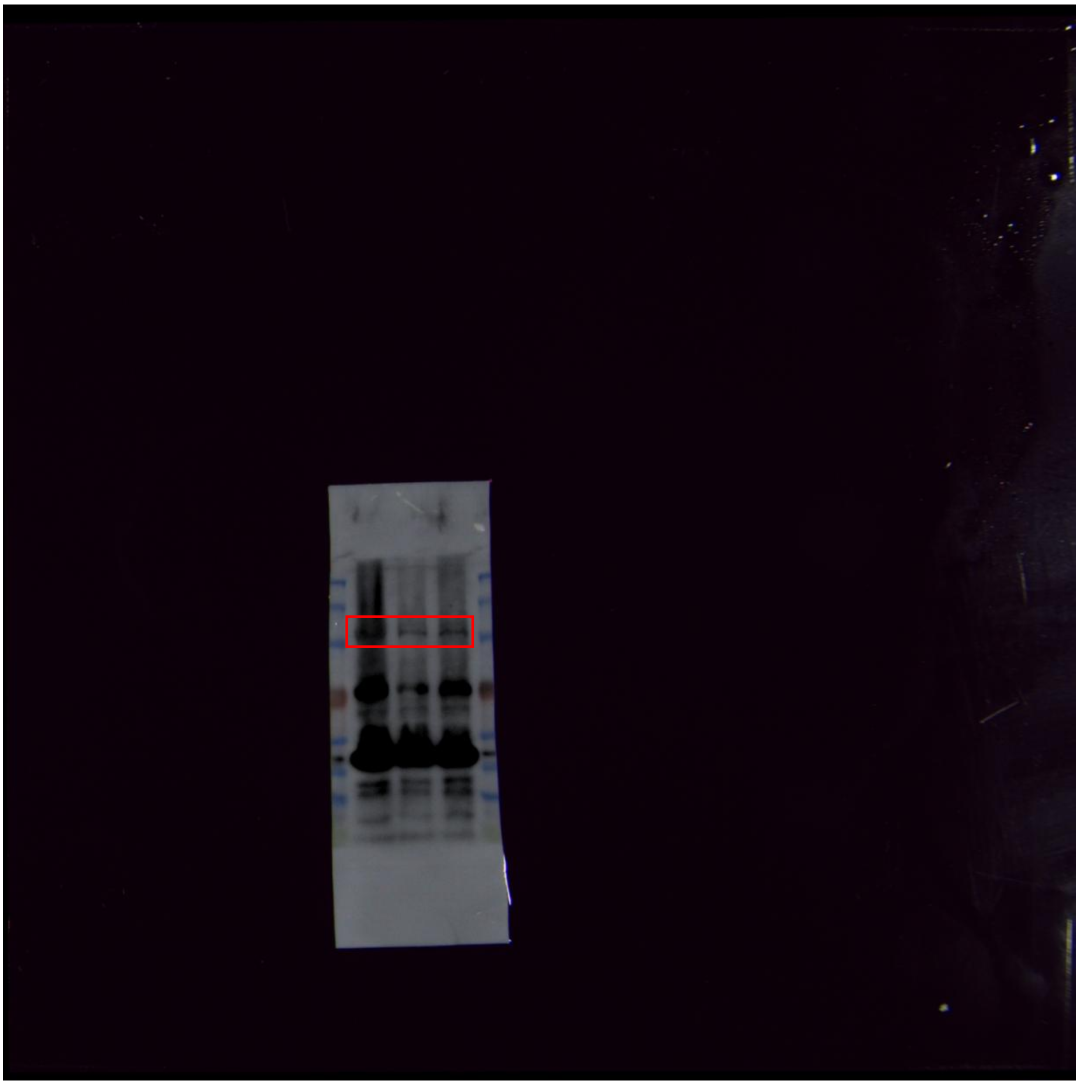

Vimentin-1:

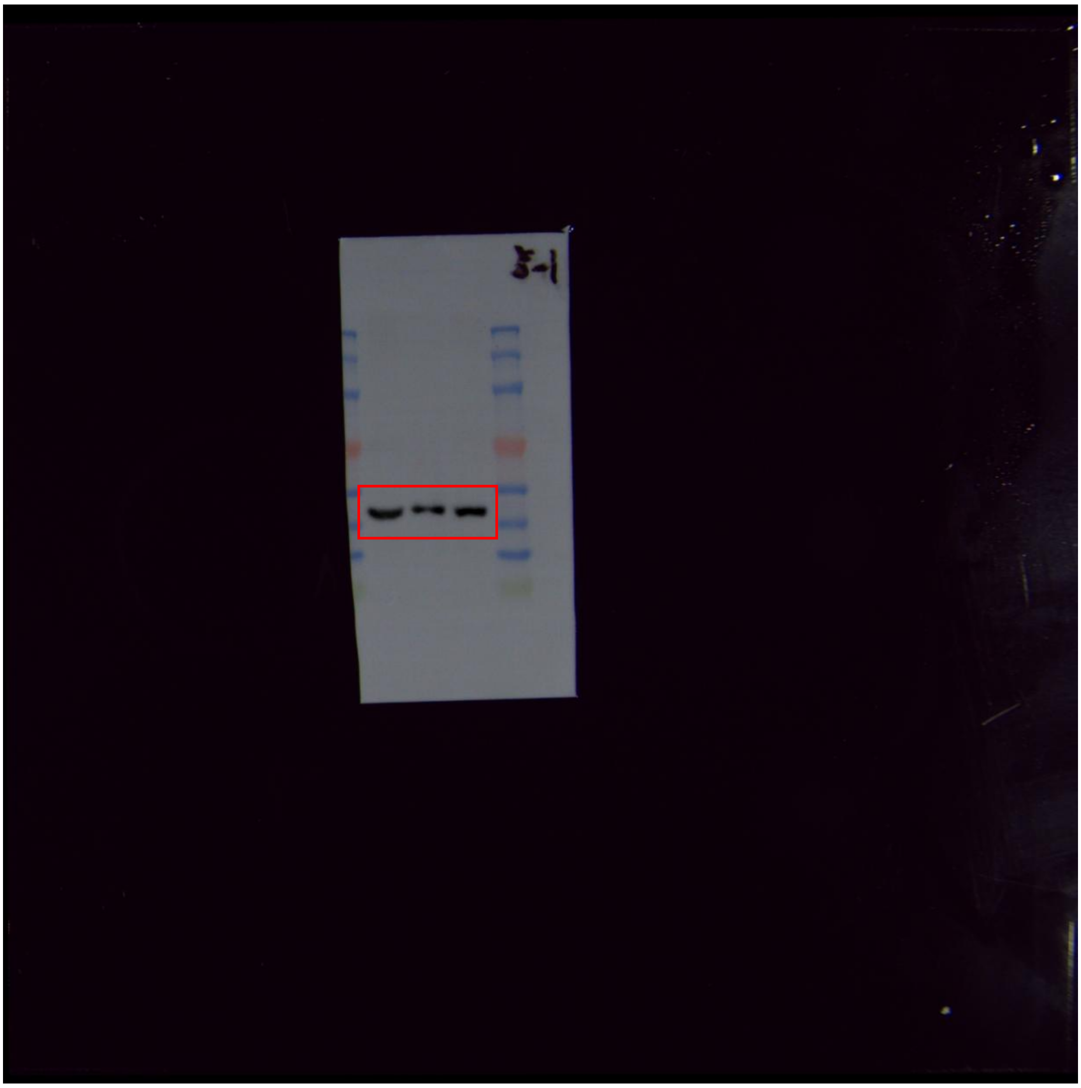

Vimentin-2:

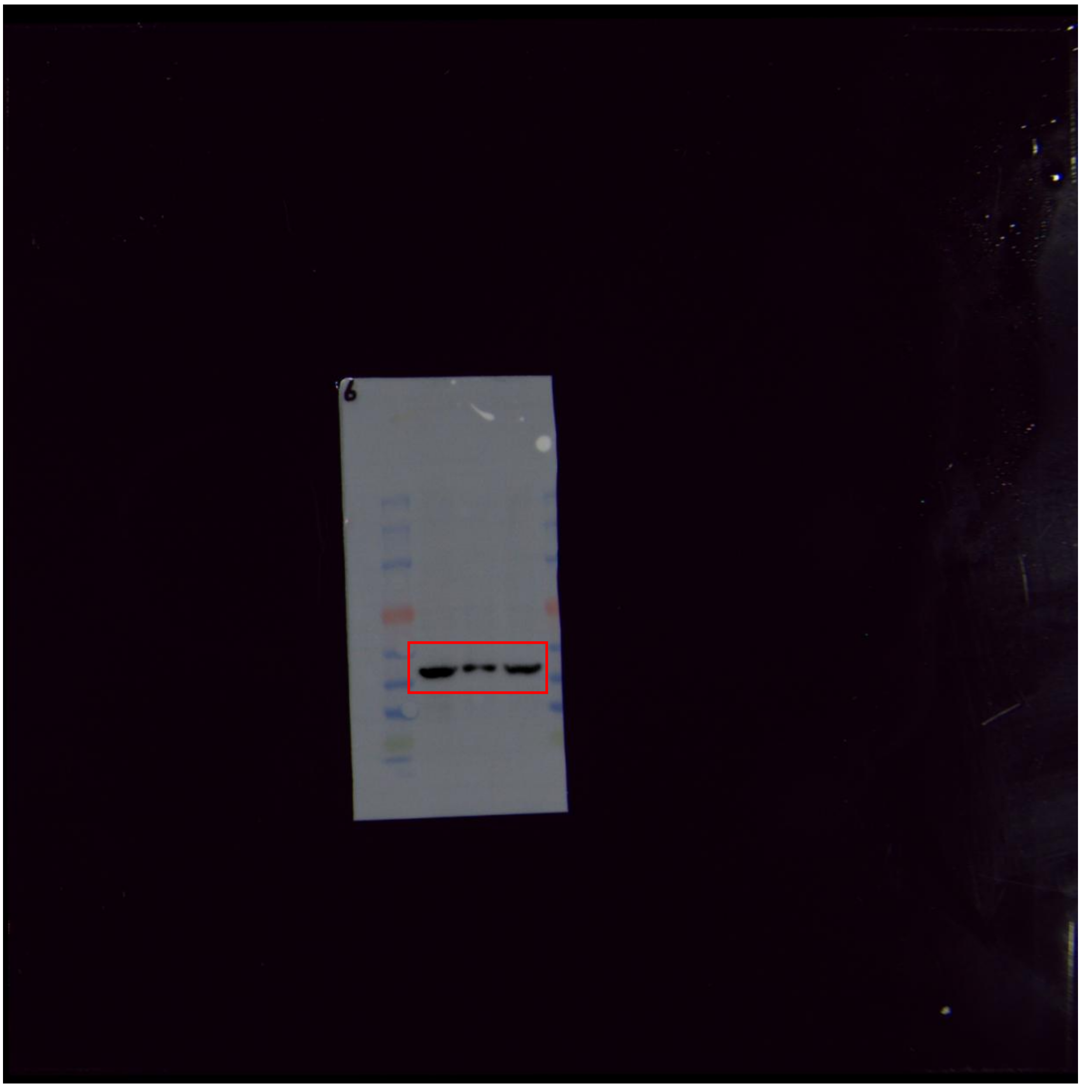

Beta-actin-1:

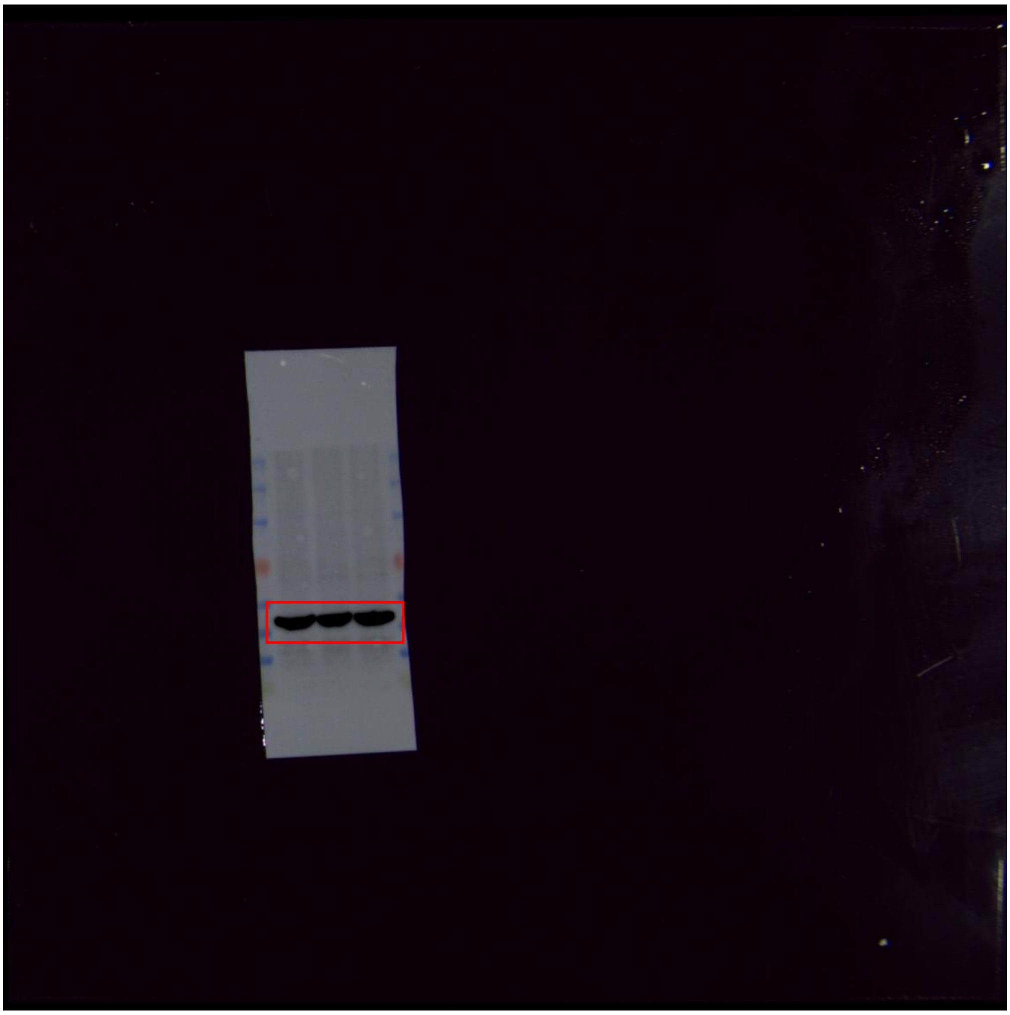

Beta-actin-2:

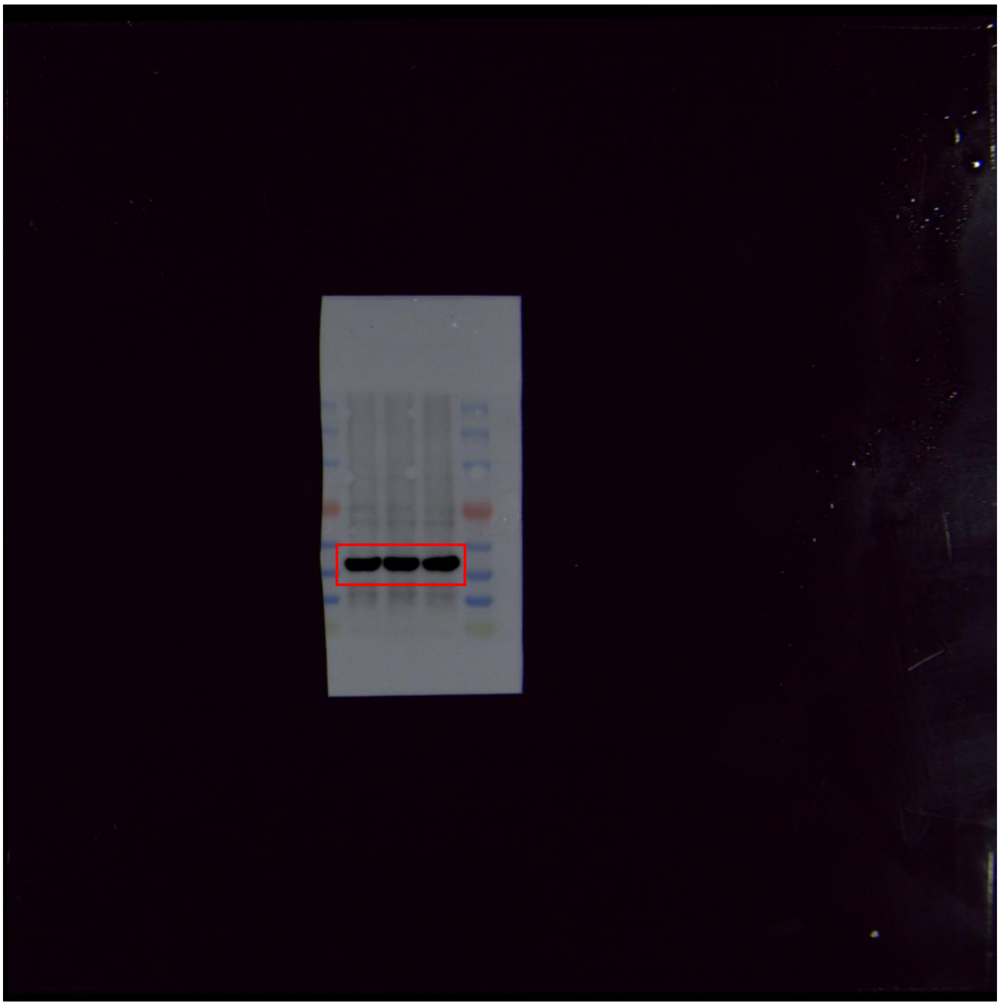

Supplement: S1 File — (PDF) [file pone.0351545.s002.pdf]
